# Understanding patient preferences, experiences and engagement with ambulatory heart rhythm monitoring: a scoping review

**DOI:** 10.1136/bmjopen-2025-110631

**Published:** 2026-05-08

**Authors:** Michael Robert James Bennett, Bill Chaudhry, Gill Norman, Tomos Robinson, Laura Ternent, Louise Coats

**Affiliations:** 1 Newcastle University, Newcastle upon Tyne, UK; 2 University of Sunderland, Sunderland, UK; 3 Sunderland Royal Hospital, Sunderland, UK; 4 Biosciences Institute, Newcastle University, Newcastle upon Tyne, UK; 5 NIHR Innovation Observatory, Newcastle University, Newcastle upon Tyne, UK; 6 Evidence Synthesis Group, Population Health Sciences Institute, Newcastle University, Newcastle upon Tyne, UK; 7 Population Health Sciences Institute, Newcastle University, Newcastle upon Tyne, UK; 8 Adult Congenital Heart Unit, Freeman Hospital, Newcastle upon Tyne, UK

**Keywords:** Adult cardiology, Telemedicine, Patient Preference, Wearable Electronic Devices

## Abstract

**Abstract:**

**Objective:**

To review the literature reporting patient preferences for ambulatory heart rhythm monitoring (AHRM) and what factors affect experience and engagement.

**Background:**

The prevalence of arrhythmia continues to rise and contributes significantly to outpatient care burden. There is limited understanding of patient experience and compliance with monitoring. As innovative technologies are developed and healthcare strategies move towards surveillance and prevention, understanding this is key.

**Methods:**

A scoping review was conducted using guidance from the Joanna Briggs Institute and reported using the Preferred Reporting Items for Systematic Reviews and Meta-Analyses extension for Scoping Reviews. The review included studies of adults under investigation or surveillance for arrhythmia with a range of devices (Holter monitor, patch device, event recorder, mobile cardiac telemetry, external and implantable loop recorders, wearables and other implantable cardiac devices) in ambulatory care settings worldwide. The final search was conducted on 3 January 2026 across Medline (PubMed), Embase (Ovid), Web of Science (Clarivate Analytics), Cumulative Index to Nursing and Allied Health Literature (EBSCOhost), PsycINFO (Ovid) and Google Scholar. Quantitative, qualitative, mixed methods, multiple methods and any type of review articles were included.

**Results:**

54 studies were eligible for inclusion from the initial search that identified 1320 articles. Two overarching themes emerged from the quantitative and qualitative data: patient factors and device factors affecting experience and engagement. Patient factors included clinical and demographic factors, education and expectations, experience and preferences and impact on daily life and healthcare. Device factors could be common to several devices, for example, skin irritation or device specific, for example, the nature of activation.

**Conclusion:**

Patient and device factors influence preferences for and experience and engagement with AHRM. While existing literature is incomplete and heterogeneous, it identifies key considerations that should be integrated into the development and testing of novel approaches for arrhythmia surveillance in healthcare contexts.

**Registration:**

https://doi.org/10.17605/OSF.IO/6K3W8 (Open Science Framework).

**STRENGTHS AND LIMITATIONS OF THIS STUDY**

- A comprehensive search across multiple databases using both keywords and indexed terms was conducted and updated immediately prior to analysis to maximise retrieval of relevant studies.
- Clear inclusion and exclusion criteria were applied using a structured Population-Concept-Context framework to support methodological consistency.
- Quantitative and qualitative data were integrated through triangulation to enhance depth and coherence of synthesis.
- The review followed established scoping review guidance and reporting standards, with pre-registration, to support transparency.

## Introduction

 Arrhythmia is a leading cause of healthcare utilisation in ambulatory and inpatient care. In 2022–2023, 2.8% of all emergency admissions in England were due to arrhythmia.^1^ Sequelae, including heart failure and stroke, contribute substantially to morbidity and mortality but can be prevented with appropriate management.^2 3^ Arrhythmia prevalence is rising, largely driven by an ageing population with higher levels of chronic disease; atrial fibrillation (AF) is the most common arrhythmia, with a current prevalence of approximately 1 in 30 adults in the UK.^4 5^

Arrhythmia care is predominantly delivered in outpatient settings and comprises monitoring, detection and treatment. However, evidence suggests the current model of care is both ineffective and inequitable.^4 6^ Oral anticoagulation and rhythm control medications remain under-prescribed, particularly in older adults and those with comorbidities, leading to missed opportunities to prevent complications.^7^,^9^ People from lower socioeconomic backgrounds are more likely to develop arrhythmia at a younger age and to die from it.^4 6 10^ Unless ambulatory heart rhythm monitoring (AHRM) can better identify arrhythmia in at-risk individuals and enable early effective treatment, cost and care burden will escalate within an already stretched healthcare system.

AHRM is the cornerstone of arrhythmia diagnosis and a range of devices are used (online supplemental file 1). Although AHRM technologies lack the detailed information provided by a 12-lead ECG, they enable prolonged recording detecting paroxysmal events, arrhythmia burden and therapy response.^11^,^13^ Traditionally, device selection has been clinician led according to symptoms or clinical risk.^14^ As clinicians acknowledge the complexity of individual health, arrhythmia management has shifted towards collaborative models actively involving patients.^15^,^17^ Current arrhythmia guidelines, such as for AF, recommend incorporating the ‘patient’s experience, values, needs and preferences’; however, little information exists on what these preferences are or their implications for designing systems of care.^13^ Despite rhetoric supporting patient centred healthcare, integration of patient preference into service design remains inconsistent and lacks established methodology.^18 19^

Patient preference is a multifaceted concept influenced by clinical, demographic and psychosocial factors.^17 20^ It is an established component of many therapeutic decisions, such as advance directives, and many diagnostics. Accommodating patient perspectives in AHRM may improve compliance, reduce health inequalities, increase arrhythmia detection enabling timely intervention and ultimately reduce morbidity.

The aim of this scoping review is to appraise and map current literature reporting patient preferences for AHRM and factors affecting experience and engagement. This is across adult populations undergoing monitoring for any cardiac arrhythmia, indication or care setting. A secondary aim is to identify evidence gaps to guide future research and implementation in arrhythmia care.

## Methods

### Conceptual framework and research question

To develop the research question, inclusion criteria and search strategies, we applied the Joanna Briggs Institution Population-Concept-Context construct.^21^ We sought to understand patient preferences, experiences or behaviours with AHRM technologies (concept) among adults (>18 years) undergoing monitoring for cardiac arrhythmia for any indication (population) in ambulatory care delivered by primary, secondary or tertiary healthcare providers in any country (context). This approach was intended to map existing evidence across devices, populations and contexts. The overarching research question was: *What do patients prefer in ambulatory heart rhythm monitoring, and what affects how they engage with and experience these devices?*

Objectives were:

- To identify patient populations and contexts studied.
- To characterise study types, devices investigated and outcomes examined.
- To identify factors impacting patient preference, experience and engagement with AHRM.
- To identify key research gaps in the literature.

### Search strategy

The initial search strategy (online supplemental file 1) was generated for MEDLINE (PubMed) and reviewed by the research team. The research question was separated into concepts to formulate keywords. Indexed terms were included alongside keywords where possible to maximise relevant results, including Medical Subject Headings in PubMed. We searched MEDLINE (PubMed), Embase (Ovid), Web of Science Core Collection (Clarivate Analytics), Cumulative Index to Nursing and Allied Health Literature (EBSCOhost) and PsycINFO (Ovid). Google Scholar (first two pages) was also searched as a supplementary platform. The original search was conducted on 8 December 2024 and updated on 3 January 2026.

### Inclusion criteria

- Adults aged 18 years or older.
- Any device used to monitor heart rhythm.
- Ambulatory care delivered by primary, secondary or tertiary healthcare providers in any country.
- Publications describing preferences, experiences or engagement with AHRM.
- Any publication date.
- Quantitative, qualitative, multiple-methods or mixed-methods research and relevant reviews.
- English language.

### Exclusion criteria

- Mixed population studies involving animals or children.
- Conference abstracts.
- Studies focused on clinician or industry preferences.

### Screening

All identified records were uploaded to Rayyan (Rayyan Systems Inc., USA) for screening and data extraction. Duplicates were removed. 50 articles were screened by two reviewers to confirm consistency in applying inclusion criteria. Remaining titles and abstracts were screened by a single reviewer; uncertainties were discussed with the wider research team. Full-text review was undertaken by a single author to confirm inclusion. Where uncertainty existed, this was resolved through discussion with a second author. Reference lists of review articles were screened to identify overlap with included primary studies; where overlap occurred, only original studies were retained. Reference lists were not screened for additional studies beyond this scope.

### Data extraction

A data collection form with prespecified variables was piloted on 10 papers and refined iteratively to ensure relevance. Data were extracted by a single reviewer and included bibliographic information, study design and methods, setting, population characteristics, device characteristics, results, discussion and study limitations. Frequencies of study characteristics were tabulated or presented graphically. Key quantitative outcomes and summary statistics were extracted. Qualitative results and discussion sections were exported as text for coding.

### Conceptual focus and synthesis

This scoping review examined patient preferences, experiences and behaviours relating to AHRM using both quantitative and qualitative evidence. Key ideas within qualitative data were identified and assigned codes; emergent themes were discussed within the research group.^22^ Qualitative themes were analysed alongside quantitative findings and synthesised through triangulation to support integrated interpretation.^23^ Findings were organised into two overarching domains: patient-related and device-related factors. Patient-related factors included clinical and demographic characteristics, education and expectations, experience and preferences, and impact on daily life and healthcare interactions. Device-related factors included features specific to individual technologies or common across multiple devices.

As this scoping review aimed to map existing literature, a level of evidence rating was applied instead of a formal quality assessment.^24^ Evidence levels were used to describe study design but not to weight conclusions, as higher-level studies often examined preference, experience or engagement as secondary outcomes, while observational and qualitative studies addressed these concepts directly. Qualitative evidence was particularly important in providing contextual and experiential insights relevant to the review objectives. This approach is consistent with scoping review methodology.^25^

### Patient and public involvement

Patients and clinicians prioritise early detection of arrhythmia as a key research area, particularly where it may inform management and reduce morbidity or sudden cardiac death.^26 27^ Prior British Heart Foundation patient and public involvement (PPI) work informed the research question and objectives of this review. There was no PPI involvement in study design or conduct.

### Registration and reporting

The study was pre-registered with the Open Science Framework (https://osf.io/6k3w8). The Preferred Reporting Items for Systematic Reviews and Meta Analyses extension for Scoping Reviews checklist^28^ was used to ensure completeness of reporting.

## Results

### Included studies

**Figure 1:**
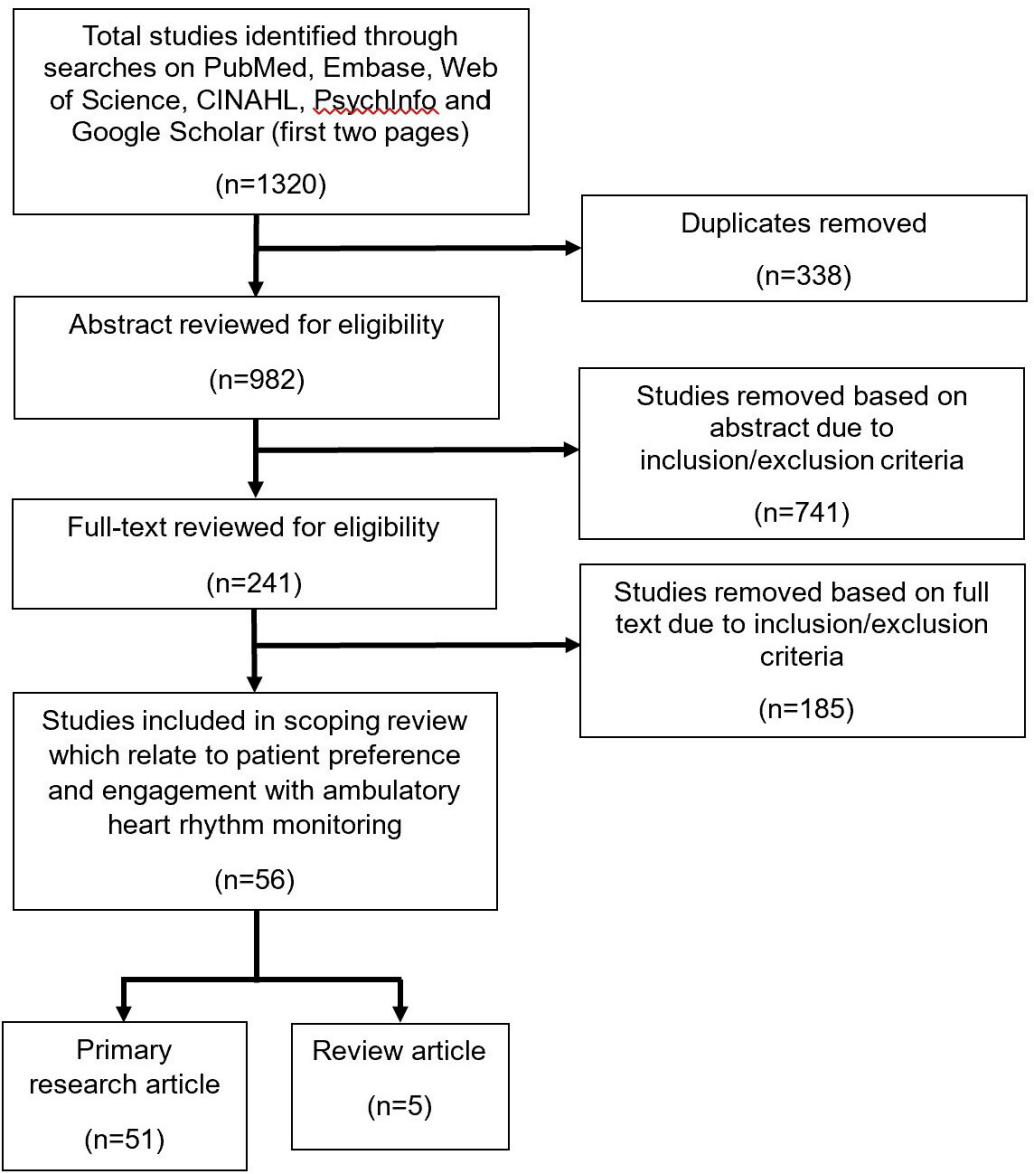
PRISMA diagram showing study selection. CINALH, Cumulative Index to Nursing and Allied Health Literature; PRISMA, Preferred Reporting Items for Systematic Reviews and Meta-Analyses.

**Figure 2:**
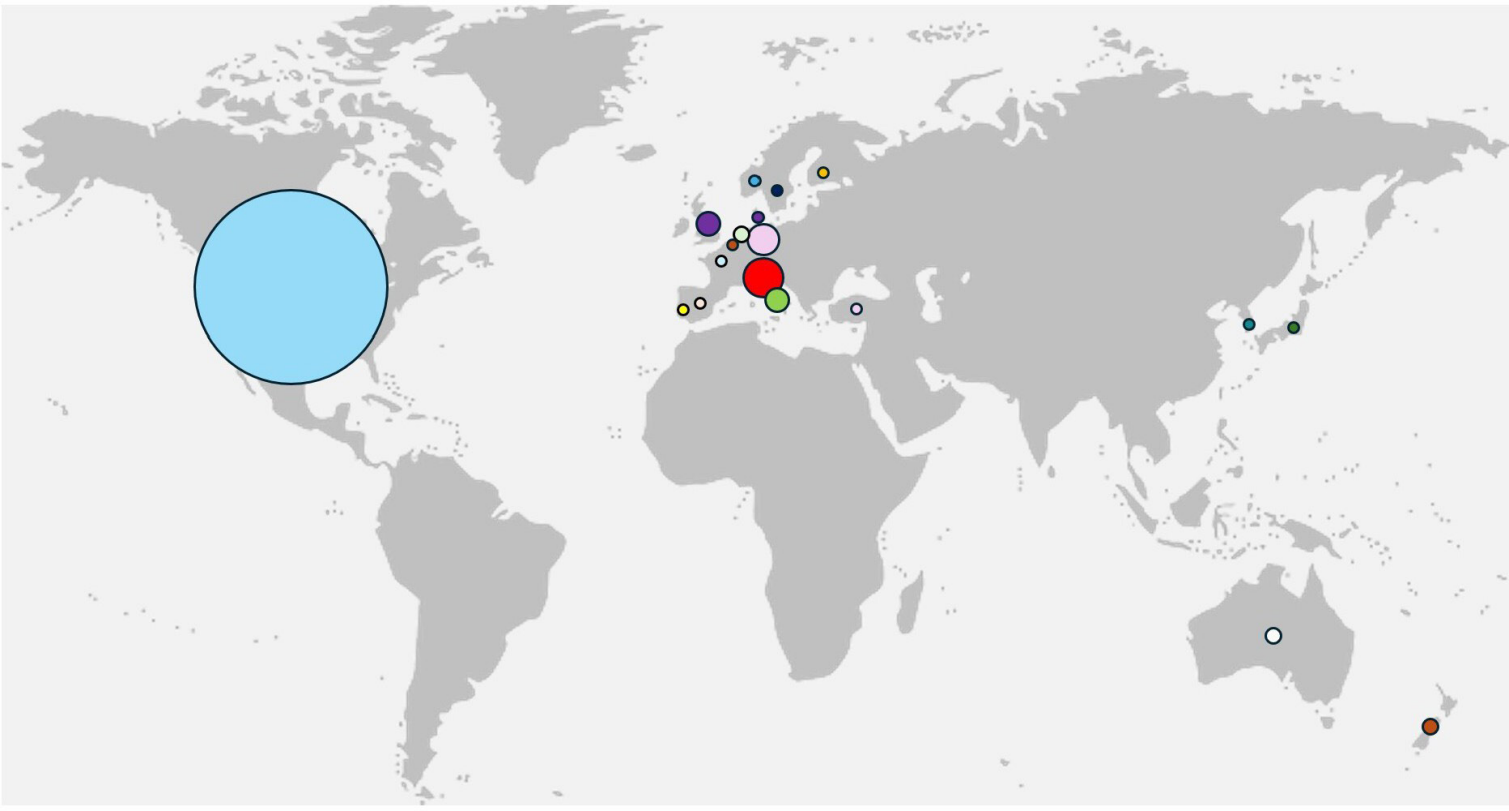
World map showing distribution of where primary research articles were performed. Each plot corresponds with the number of studies performed in that given country (range=1–25).

The initial search identified 1320 studies. Following removal of 338 duplicates, 982 abstracts were screened. After exclusions, 241 full texts were assessed and 54 studies were included (figure 1). Of these, 49 were primary research and 5 were reviews (online supplemental file 1). All primary research studies were conducted in high-income countries, predominantly in ambulatory settings supervised by secondary or tertiary care providers (96.1%) (figure 2).

### Populations and context

The 49 primary research studies (2008–2026) included 7155 patients (mean age 62.2 years; range of mean ages 33.0–81.9; 36.7% female). Follow-up duration was a median of 34.7 days (range 1–730).

Studies reported on Holter monitors (13 studies, n=1081), patch devices (18 studies, n=1911), event recorders including smartphone-based systems (13 studies, n=853), mobile cardiac telemetry (3 studies, n=315), external loop recorders (2 studies, n=215), implantable loop recorders (ILRs, 14 studies, n=1072), wearable devices (7 studies, n=1460), smartwatches (7 studies, n=2470) and implantable cardiac devices, including permanent pacemakers (PPMs) (5 studies, n=507) and implantable cardioverter-defibrillators (ICDs) (8 studies, n=1490). Studies involving ICDs and PPMs were included only if they reported on patient preference, experience or engagement with these devices for remote monitoring purposes.

**Figure 3:**
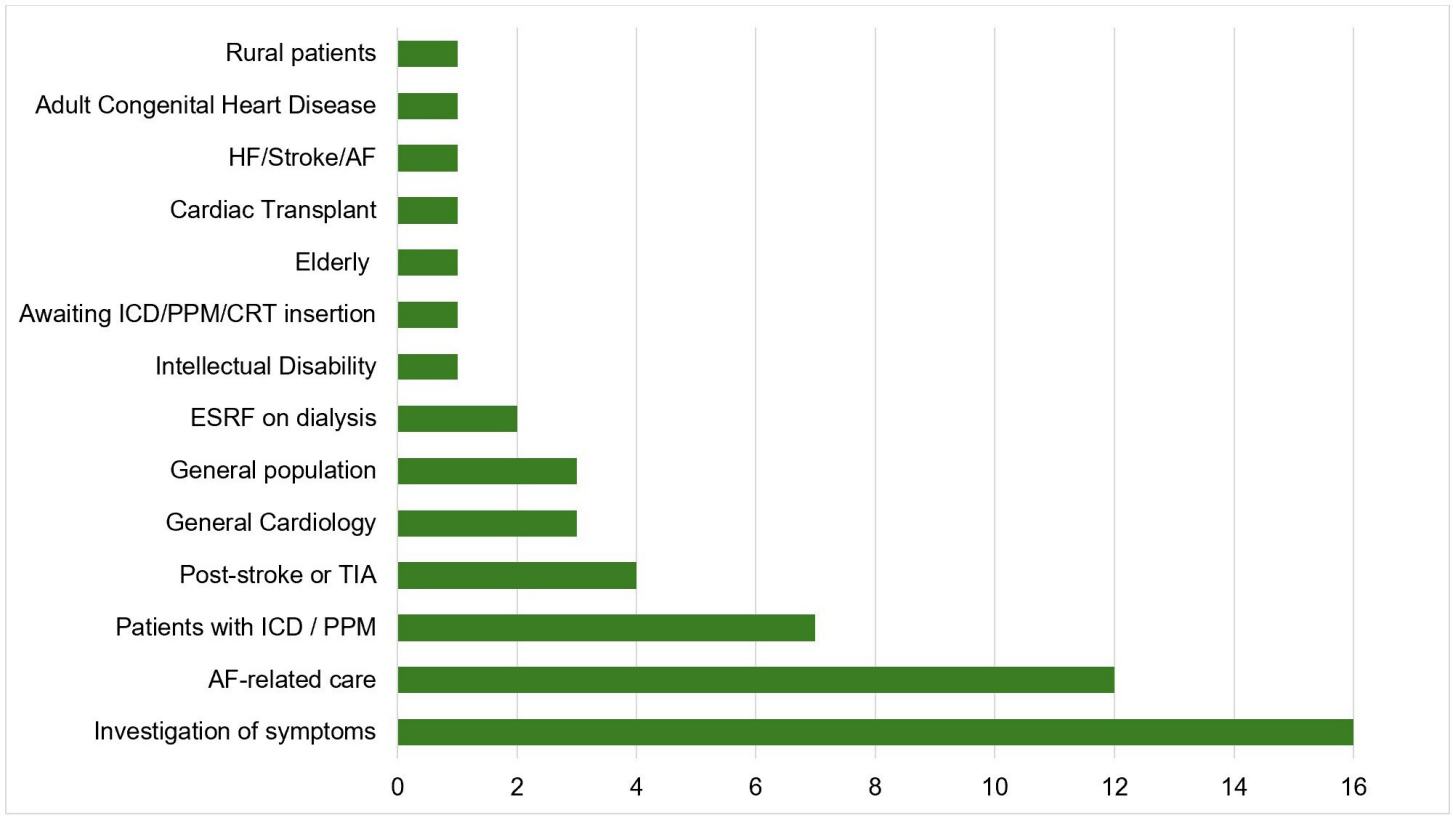
Distribution of included studies by patient population receiving ambulatory heart rhythm monitoring. AF, atrial fibrillation; CRT, cardiac resynchronisation therapy; ESRF, end-stage renal failure; HF, heart failure; ICD, implantable cardioverter defibrillator; PPM, permanent pacemaker; TIA, transient ischaemic attack.

Monitoring was primarily performed for investigation of symptoms (16 studies, n=2048) or AF-related care (12 studies, n=1430). Specific cohorts included patients with end-stage renal failure (two studies, n=55) and those with recent cerebrovascular events (four studies, n=235) (figure 3).

### Methodology of included studies

Primary research articles were quantitative (n=31), qualitative (n=7), multiple methods (n=8) or mixed methods (n=3) (online supplemental file 1). Mixed-methods studies were defined as those where qualitative and quantitative data were integrated at design or analysis stage, in contrast to multiple methods where both data types were collected but not formally integrated. Quantitative studies collected information through questionnaires (n=29) or observational metrics such as patient compliance (n=19). Questionnaires used binary, stated-preference or Likert-scale responses. Qualitative studies used focus groups (n=2) or semistructured interviews (n=5). Mixed-methods or multiple-methods studies used free-text boxes (n=8) or interviews (n=3). Validated questionnaires were used in eight quantitative and four mixed-methods/multiple-methods studies and assessed self-perceived physical and mental health, AF burden, mobile health usability, medication adherence, ICD acceptance, control of cardiac disease and technology acceptance (online supplemental file 1). No studies employed formal stated-preference methods (eg, discrete choice experiments or contingent valuation).

### Themes and interpretation

Two overarching themes emerged: patient factors affecting monitoring and device factors affecting monitoring. Subthemes were generated through triangulation of quantitative and qualitative data to integrate findings and identify patterns (table 1 and table 2). Mapping of individual studies to specific subthemes is provided in online supplemental file 1.

**Table 1:** Joint display table presenting quantitative and qualitative data for patient factors affecting preference, experience and engagement with ambulatory heart rhythm monitoring

Theme	Quantitative (device specified)	Qualitative (device specified)	Interpretation
**Clinical and demographic factors**			
Age	Event recorder: use increased with age (OR 1.06; 95% CI 1.01 to 1.11).^33^ iPhone ECG: no age effect in post-surgery cohort.^31^ ILR: age unrelated to transmission failure.^69^ ICD/PPM: age did not affect cooperation with remote monitoring (p=0.83),^58^ although older age was linked to reduced patient engagement with ICD monitoring processes.^35^ Smartwatch (stroke/TIA, n=104): no age differences in anxiety or wear time.^98^	Holter/smartwatch: ≥75 years declined participation due to smartphone compatibility or complexity—“I don’t have a smartphone that works with it”.^73^	Age does not appear to impact the ability to comply with rhythm monitoring strategies, but evidence is limited.
Comorbidity	Patch ECG: intellectual disability associated with shorter wear (9.2 vs 10.8 days; p<0.05).^37^ Patch ECG (AF detection, n=256): higher BMI predicted poorer compliance (B=−0.046; OR 0.955; p=0.03).^99^ ICD: comorbidity/LV dysfunction reduced data transmission.^34^	ILR: authors suggest that patients with ESRF who declined ILR may feel ‘overburdened’, rather than ‘non-compliant’.^29^ ILR insertion post-cardiac surgery was some declined by 24% of patients (14/58), as they felt ‘overwhelmed’.^63^	Monitoring burden may exceed coping capacity in multimorbid or cognitively impaired groups.
Symptoms	Event recorder (palpitations): frequent symptoms predicted sustained use (OR 1.42; 95% CI 1.07 to 1.89).^33^	Event recorder (KardiaMobile): some patients contacted clinicians rather than increasing recordings when symptomatic.^36^	Symptoms prompt increased usage of monitoring but those who disengage revert to standard reporting.
**Education and expectations**			
Understanding of arrhythmia	Event recorder (post-op AF, n=42): AF knowledge improved (6.4±1.8 to 7.3±1.8; p=0.02) with remote monitoring.^31^ ICD (n=342). ICD: 86% (n=342) demonstrated knowledge deficits about their device.^38^ Patients demonstrated comparable knowledge of most monitoring devices across countries (UK, Germany, USA, Japan), but awareness of mobile cardiac telemetry varied, reflecting greater availability in Europe and the USA.^54^	ILR (ESRF): “I never felt it happen…my heart would go from like 80 beats a minute up to over 130. That’s the arrhythmia that they discovered, but I didn’t even know I had it”.^29^ Authors conclude patients with ESRF did not realise their symptoms were attributable to arrhythmia.^29^ Patients report receiving little information about how their life could be affected by monitoring and wanted more.^36 45^	Monitoring can improve knowledge, but baseline understanding is often limited. Provision of information by clinicians is not always done well.
Expectations and concerns	ILR: 60% (18/30) feared next event post-implantation.^46^ Prior ECG familiarity correlated with perceived monitoring need (r=0.35–0.48; p<0.001).^43^	ILRs: prior to monitoring, patients report a ‘loss of independence or security’.^53^ In those ESRF, they were concerned about its effect on airport security, tracking, malfunction.^29^ Holter/smartwatch: patients were unsure what symptoms may justify recording an ECG—“So you have to make a decision yourself: do I make a recording or not?”^73^ and reported they would prefer a smartwatch if it was equally as effective—“If those watches pass the trial and give good results compared with that recorder, then that would actually be much easier”.^73^	Patients have a wide range of practical concerns with rhythm monitoring and uncertainty generates anxiety. Prior usage and knowledge increase perceived need for monitoring.
**Experience and preferences**			
Experience of monitoring	Event recorder (HF): 85.5% (n=288) reported improved confidence with use.^39^ ILR: 60–70% felt safer; ~20% felt sicker over 6 month period of use.^47^ Smartwatch: in patients who had a stroke (n=96), smartwatch ownership was associated with improved physical health (SF-12, p<0.05) but not anxiety (GAD-7).^100^ In AF patients (n=172), smartwatch users reported greater symptom monitoring (p=0.03), increased treatment concerns (p=0.02) and 20% experienced anxiety with frequent clinician contact following alerts.^83^ ICD/PPM: 92% (n=119) reported sense of security and 95% reported positive impact on health through arrhythmia monitoring.^59^ In a separate study of ICDs (n=343), issues occurred like 28% made unscheduled transmissions (34%), shocks (19%) and alarms (19%).^76^	Event recorder: “It was absolutely fantastic because I would never have been able to keep a track of what was happening”.^31^ ILR: “It does give me that feeling like something is watching all the time, like 24/7 diagnostic”.^45^ Confirmation of arrhythmia led to relief but symptoms with normal recordings led to frustration.^44^ Patch device: one patient reported that wearing a patch device for syncope increased ongoing worry about recurrence, which they felt would not have occurred without the device.^52^	While quantitative data shows no effect or improved experience with monitoring, qualitative findings identify that some individuals experience additional uncertainty. When discordance between symptoms and recordings arise, new anxieties are introduced.
Preference and compliance	Event recorder: use of KardiaMobile (n=33) was higher than external loop recorder (91.2% vs 52.7%; p=0.01) to investigate palpitations.^60^ 73% (23/31) of heart transplant patients did daily ECGs for 3 months,^64^ and 86% (36/42) of post-cardiac surgery patients recorded >3 ECGs/day for 27 days using an iPhone ECG.^31^ Patch versus Holter: 85% (159/187) patients prefer patch devices to Holters^58^ and 92% (72/78) prefer event monitors to Holters.^70^ Smartwatch versus ILR: willingness for long-term use was highest for Apple Watch 6 and Fitbit Sense (3.8) and lowest for KardiaMobile (3.2).^55^ AF patients showed higher wearable ownership (n=1222).^101^ Adoption was positively associated with performance expectancy (β=0.205), habit (β=0.192) and innovativeness (β=0.185), but not privacy or accuracy.^56^ In congenital heart disease (n=9), adherence was low (~25% expected ECGs over 4 months).^102^	Patients preferred less invasive and commercially familiar devices.^73^	Patients prefer less invasive and data suggests a preference for commercially available devices over hospital prescribed ones. Over three quarters of patients can comply with self-monitoring requirements.
Ease of use	Event recorder: 98.6% HF and 95% post-surgery found easy to use.^31 39^ Event recorder versus external loop: easier to use (1.4 vs 2.7; p<0.01).^60^ Smartwatch: recording affected by motion, temperature, tattoos.^74^	Event recorder: “I don’t have those sorts of iPhones and things, but I handled it quite well and didn’t have any problems”.^31^ “On weekends I didn’t do it…from the beginning I wasn’t doing it every day. I guess, I just forgot it. I don’t take it to work”.^36^ Authors report device is “easy to use with minimal, if any, learning curve”^36^ and that HF patients with less prior technology use required more support from family and clinical staff.^44^ Poor ECG tracing due to improper finger placement, forgetting to charge and insufficient education were cited as issues.^31 53^ Smartwatches: a patient found the device could miss symptoms–“it skips for me and by the time I press it, that skipping is already gone.”^73^	Usability was high across devices. Patients generally managed unfamiliar technology without increased anxiety, benefiting from clinical or personal support. Common issues included forgetting to record or transmit data and insufficient education.
**Daily life and healthcare interaction**			
QoL/ADLs	Holters: ADL impact 44% versus 4% with patch (p=0.01); sleep disturbance 42% versus 8% (p<0.01),^58^ Patch ECG: 74% could shower while wearing;^50^ less impact on symptoms and physical functioning than external loop recorders (p<0.01).^57^ Event recorders: preferred over external loop recorders in work/social settings (p<0.01);^60^ no difference in AF QoL versus control (79 vs 82; p=0.61).^32^ ILRs: 97% reported no QoL impact (29/30).^46^ ICD/PPM: 10% reported daily activity impact; 5% found monitoring a ‘bother’ over 1 year.^59^	Patch ECG: facilitated exercise better than Holter, including “football games, outdoor jogging, aerobics and similar activities”;^41^ described as having “no disadvantages whatsoever” and that “you more or less forget about it”.^52^ Holters: caused skin irritation and inconvenience—“the wires get caught on something - that’s just annoying”.^73^ ILR: led to changes in daily routine and “personal and social identity”.^44^ Smartwatches: considered minimally burdensome—“you barely notice you’re wearing it not even at night”.^73^	Holter most disruptive and affects normal routines including sleep. Patch and ILRs less intrusive. Monitoring is more easily incorporated in the home environment than work or social situations.
Healthcare usage	Holters: required more time for collection and return than other devices (n=21; p<0.001).^61^ Event recorders (KardiaMobile): no change in healthcare utilisation post-AF ablation (p=0.04).^62^ Smartwatches: higher AF-related healthcare use over 9 months (n=172; p=0.04), including greater informal contact (telephone/portal messages; p=0.05).^83^	ILRs: perceived as reducing hospital visits—“Because it is efficient and convenient so you will not have to come to the hospital in person more often”.^43^ ICD/PPM: remote monitoring reduced face-to-face contact for some, while others preferred fewer in-person clinic visits.^76^	Patients feel they may experience less healthcare use with rhythm monitoring however there is insufficient evidence to support this.
Relationship with healthcare professionals	Event recorders: 52.6% (120/288) of patients with heart failure reported improved clinician contact with remote monitoring.^39^ ILR: no preference for nurse-led versus physician-led follow-up;^39^ insertion by nurse (n=189) or clinician (n=132) equally successful (100%) with high satisfaction (99% vs 97%).^40^ Smartwatches: AF patients more likely to share data with clinicians than at-risk individuals (OR=1.87; 95% CI 1.0 to 3.4).^101^ ICD/PPM: 97% (115/119) reported positive clinician relationships;^59^ 13% (45/346) sought consultation for health/device queries and 39% (115/295) felt GPs were insufficiently informed;^76^ 90% (270/300) desired direct clinician communication alongside data transmission.^75^	Holters: patients happy for follow-up to shift from cardiologist to GP if it reduced waiting times.^41^ ILRs: patients with ESRF preferred doctors to provide device education.^29^ Event recorders: remote monitoring enhanced perceived connection—“I feel like I am… 99% in tune with them, or they with me, because it just gives them such important information”.^36^ ICD/PPM: some reported difficulty obtaining information—“The information I know about the device… are because I asked a ton of questions… I’d like the information as a matter of course with a good conversation. It is (like) pulling teeth.”^38^ others needed reassurance—“you need a human, or at least I need a human connection somewhere in the year to reassure me and support me in having this thing”.^67^	Patients experience an improved bond with healthcare professionals when undertaking remote monitoring. They want information to be provided by doctors but report satisfaction with and acceptance of other healthcare professionals delivering and overseeing care.
Feedback of results	Event recorders: 84.6% (244/288) of patients with heart failure wanted 24-hour clinician access.^39^ ICD/PPM: patients requested detailed feedback—battery life (84%, 287/342; 83%, 249/300), activity (79%), heart rate trends (75%), ventricular arrhythmias (74%), device function (82%), lead integrity (81%), heart rate variability (90%) and arrhythmia occurrence (62%).^38 75^	Event recorders: perceived imbalance in communication—“It seemed like a one-way street where you guys were just taking my information and I’m out there on my own”;^36^ infrequent use led to “confusion and difficulty interpreting the data”.^36^ ILRs: reassurance was valued—“They would call me immediately, ‘cause they said they monitor these things constantly”.^45^ Smartwatches: false positives generated anxiety; unclear automated ECG outputs contributed to uncertainty.^73 74^ ICD/PPM: patients requested transparency—“I know my body! The more information I have…the better!” and “should be given the option to know everything about the pacemaker”.^38^ Some wanted immediate confirmation of transmission receipt and results:^76^ “Do I wish I had more information about it? Yes…I don’t know if somebody’s looking… Because I hear nothing”.^67^ Patch ECG: device failure could undermine confidence—“Then after about 13 days, the battery actually failed, it just ran out of battery.”^52^	Healthcare provider feedback was important to patients, but frustrations arose when the focus was on the data rather than the experience of the individual. Where remote monitoring devices are mandated for therapeutic purposes, patients consider they receive insufficient information from them.

ADLs, activities of daily living; AF, atrial fibrillation; BMI, body mass index; ESRF, end-stage renal failure; GAD-7, Generalized Anxiety Disorder 7-item Score; GP, general practitioner; HF, heart failure; ICD, implantable cardioverter defibrillator; ILR, implantable loop recorder; LV, left ventricular; PPM, permanent pacemaker; QoL, quality of life; SF-12, Standard Form Health Survey; TIA, transient ischaemic attack.

**Table 2:** Joint display table presenting quantitative and qualitative data for device-related factors affecting preference, experience and engagement with ambulatory heart rhythm monitoring

Theme	Quantitative (device specified)	Qualitative (device specified)	Interpretation
Complications	Holter monitors and patch ECGs: 60% (12/20) removed devices early due to skin irritation.^50 57 58 72^ ILRs: less pain (p=0.019) and better wound healing (p=0.003) but more bruising (p=0.041) when inserted by a nurse versus doctor.^40^ A minority experienced persistent pain, discomfort or paraesthesia,^40 46^ and device malfunction and MRI interference also contributed to early explantation.	Event recorders: skin irritation from the electrodes was reported as the main issue.^53^ ILRs: authors report, “Participants described physical, cognitive and emotional symptom changes that were different after the ILR was inserted that influenced the way they self-managed”.^45^	Skin irritation with external devices and pain with implanted devices affect a significant proportion of patients and impacts how patients self-monitor and can lead to discontinuation of monitoring.
Comfort	Holters and patch ECGs: one study found no comfort difference (1.0 vs 2.9, Likert 1–5; p=0.063) in 21 AF patients.^61^ Two studies reported patch ECGs were more comfortable than Holters.^51 58^ 94% (31/33) found patch ECG comfortable for 14 days.^50^ External loop recorders and patch ECGs: patch ECG comfort was 98% (41/42) versus 90% (18/20) for external loop recorders (p=0.16).^57^	Holters and patch ECGs: one patient described Holter as a “living medical instrument”; in contrast, a patch was preferred as “the wireless sensor was comfortable to wear and most of the time I forgot I was wearing it”.^41^ Smartwatches: device choice was driven by “design, followed by a pre-existing familiarity” rather than “comfort, ease of use, simplicity, or handiness”; issues included “wristbands not having a traditional belt buckle”.^55^	Newer devices (patch ECG) are generally more comfortable than traditional Holter monitors but patients expressly choose smartwatches for their design and comfort.
Cosmetic	Holters and patch ECGs: Holters were perceived as visible by 28% (14/50) versus 12% (6/50) for patch ECG.^58^ ILRs: 93% (28/30) reported satisfactory cosmetic outcome.^46^	Holters: patients reported stigma, wanting to “hide a cardiac monitor from the general public”; activating the event button could be socially awkward—“Especially as a woman, it’s a bit less fun to pull up your shirt.”.^73^ Patch ECG was perceived as more discreet.^103^ ILRs: patients with ESRF expressed cosmetic concerns—“would I be able to see a big knot there?”.^29^ Patch ECG: “I had to wear a higher neck top to hide it. Once it was on after a couple of days, I just became used to it.”.^52^	Patients are aware of the visibility of monitoring devices and qualitative data suggest that this is associated with stigma.
Data transmission and privacy	Event recorders: 18% ECG data loss due to failed transmission in heart transplant patients;^64^ rural post-cardiac surgery patients reported mobile connectivity-related data loss.^31^ ILRs: only 24% (17/70) post-cardiac surgery patients (mean age 72) transmitted data independently without telephone or hospital support;^69^ in a symptomatic cohort (n=33), transmission was reported as fast and easy.^47^ Implantable cardiac devices (ICD/PPM): 91% (54/59) found data transmission easy without assistance.^34^	Smartwatches: patients reported “concerns of data safety”, including data protection and private company involvement, influencing willingness for long-term use.^55 74^	Patients are concerned about the security of their data Information can be lost at transmission and may exacerbate health inequalities.

AF, atrial fibrillation; ESRF, end-stage renal failure; ICD, implantable cardioverter defibrillator; ILR, implantable loop recorder; PPM, permanent pacemaker.

Of the included studies, 48 reported patient-related and 33 reported device-related factors. Patient factors (table 1) addressed impacts on activities of daily living and healthcare interaction (36 studies); experience and preferences (34 studies); education and expectations (16 studies) and clinical and demographic factors (15 studies). Device-related findings (table 2) concerned specific device characteristics (31 studies) and general device factors (19 studies).

### Patient factors

#### Clinical and demographic factors

Patients with chronic comorbidities demonstrated additional challenges in engaging with monitoring. Patients with end-stage renal failure, despite high arrhythmia prevalence, often attributed symptoms to renal disease, delaying diagnosis until emergency presentation.^29 30^ They expressed concerns that ILRs might interfere with dialysis treatment, stating “I’m sure it might complicate the catheter”.^29^ AHRM may therefore increase burden of care beyond what some patients can easily manage.

Older adults benefitted from training and support and, despite limited technological experience, were often consistent users. Event recorder use increased with age (OR 1.06; 95% CI 1.01 to 1.11), and no age-related differences were seen in iPhone ECG use following cardiac surgery.^31^,^33^ However, older adults were less likely to activate ICD remote monitoring when required, highlighting device-specific engagement challenges.^34^

Symptoms increased use of monitoring:^33 35^ “I probably use it too much because every time I have chest pain, I just pull it out”.^36^ However, where symptoms occurred but arrhythmia was not demonstrated, subsequent use declined.^36^

Health inequalities were evident. Rural patients experienced data loss of up to 18% of transmissions, and patients with intellectual disabilities wore devices for significantly shorter durations (9.2 vs 10.8 days; p<0.05).^31 37^

#### Education and expectations

Although many patients with cardiac conditions had baseline knowledge of rhythm disorders, premonitoring education was often perceived as insufficient. In one study, AF knowledge improved from 6.4±1.8 to 7.3±1.8 (p=0.02) following targeted information, while another reported that 86% of ICD patients demonstrated key knowledge gaps.^31 38^ Patients preferred information from doctors but accepted other healthcare professionals delivering and overseeing care.^39^,^41^

Patients expressed concerns regarding device safety at airports, potential tracking and data privacy. AHRM sometimes improved knowledge about arrhythmia but could perpetuate perceived need for continued monitoring. Monitoring was viewed as a step towards answers,^3642^,^44^ a ‘defining moment’^42^ or a ‘time to make a change’.^36^ Authors observed that “individuals are living with the hope that the cause of their symptoms will be discovered”.^45^

#### Experience and preferences

Arrhythmia symptoms caused distress, disrupted daily life and threatened “loss of independence or security”.^42 45^ Overall, AHRM did not compromise well-being and often created a ‘feeling of safety and being free of worry’.^45^ However, a minority experienced increased uncertainty. While 60–70% of ILR patients felt safer within 6 months, around 20% felt sicker or more anxious, particularly when symptoms persisted despite normal recordings.^46 47^ “The uncertainty is the worst part. It can eat you up from the inside…all you can do is wait for an attack or episode to happen before you can get any diagnosis”.^44^ ILR insertion provided security for some but represented invasion of privacy for others.^4046^,^48^

Patients generally preferred less invasive devices. Between 81 and 92% favoured patch or event monitors over traditional Holters, and 98% of patients who had a stroke preferred smartwatches over ILRs.^49^,^51^ Most technologies were considered easy to use, particularly if familiar. Support from family and friends helped when monitoring was a new experience.^44^ Written information was valued for reinforcing verbal explanations, though some patients found terminology too complex to support self-management and troubleshooting.^52^ Where a prescribed device failed to diagnose arrhythmia, patients preferred alternative options if offered.^42^ Previous monitoring experience therefore influenced future device preference.^29 43 44^

Compliance was generally high across devices (>75%). However, up to one-quarter of patients forgot to activate or charge devices regularly, particularly with event recorders and smartphone-linked technologies.^31 53 54^ In smartwatch comparisons, brand familiarity strongly influenced preference.^55^ Another study found smartwatch use driven more by perceived usefulness, habit and technology literacy than by health orientation or accuracy beliefs.^56^

#### Impact on daily living and healthcare interaction

The impact of AHRM on activities of daily living represents an important factor influencing patient preference, experience and engagement with monitoring. AHRM had potential to impair quality of life by restricting activities such as swimming, showering, exercise, sleep and work.^46 51 53 57 58^ This affected ‘personal and social’ identity.^44^ Holter monitors were more disruptive (44% reporting activity of daily living impact vs 4% with patches; sleep disturbance 42% vs 8%, p<0.01), whereas ICD monitoring affected only 5–10% of patients, though could increase anticipatory anxiety.^50 59^ Monitoring was more easily incorporated at home than in work or social environments.^44 60^ Some patients integrated event recorders into daily routines, using them at bedtime or meals.^31^

Patients perceived reduced healthcare attendance with AHRM: “it is efficient and convenient so you will not have to come to the hospital in person”.^43^ Quantitative studies showed mixed results on whether monitoring reduced attendance.^61^,^63^ Remote transmission saved travel time, but data upload could be time consuming and subject to loss if performed incorrectly.^31 47 64^ More than half of patients with heart failure reported improved contact with clinicians, and 84.6% wanted access to 24-hour feedback.^39^ Patients placed high trust in clinicians when deciding on ILR insertion: “They recommended [it], who am I to say; He [clinician] suggested it and I agreed with it”.^42^ However, some felt their personal experience was overlooked in favour of ECG data:^36 44 65 66^ “it seemed like a one-way street where you guys were just taking my information and I'm out there on my own”.^36^ Frustration was expressed where care teams lacked sufficient knowledge to answer questions about implanted devices or remote monitoring: “My cardiologist really seems to have no connection to it at all… I was surprised*”*.^67^

### Device factors

#### General factors

Each remote monitoring device generated a distinct interaction with patients.^40 46 48 68 69^ Holters and ILRs required minimal patient involvement once fitted, while event recorders and wearables required patient activation; nevertheless, devices were generally regarded as accessible and user friendly.^32 39 43 53 60 64 70^ A common issue across devices was skin irritation from electrode contact,^50 53 57 58 61 71 72^ leading to early removal in up to 60% of cases.^71^ Concerns regarding data security and transmission failure were also reported, particularly among rural populations experiencing up to 18% data loss.^64 71^

#### Specific factors

Holter monitors were criticised for size and visibility,^41 58^ with one patient describing feeling like a ‘living medical instrument’.^41^ They were consistently less preferred than alternative AHRM devices. Patch ECG devices were often preferred,^41 50 58 70 72^ described as “comfortable to wear and most of the time I forgot I was wearing it”.^41^ Reasons for preferring patches included being lightweight, wireless, water resistant and discreet.^41 50 58 70 72^ Patients found them easy to apply and tolerated them over longer periods.^57 61^

Event recorders, including KardiaMobile, required patient activation and could be forgotten.^49 53^ Difficulties included charging, smartphone app interaction and poor finger placement, leading to non-diagnostic tracings, particularly in patients with tremor^31 36 65 66^

ILRs were the only devices without therapeutic function requiring an invasive procedure.^40 48^ Premature removal occurred due to pain, infection, malfunction, MRI incompatibility, paraesthesia or patient request.^40 46 48 68 69^ Qualitative data revealed concerns among dialysis patients, one stating “NO more battle scars” regarding insertion scars.^29^

Smartwatches demonstrated broad acceptance, with design a key factor.^55^ One qualitative study suggested that trust in device reliability may be shaped by perceived ‘medical’ versus ‘commercial’ appearance, with one participant stating “the smartwatch looks more like a commercial product and the Holter monitor more like medical equipment,” implying lower trust in the smartwatch.^73^ Overall preference nonetheless favoured smartwatches, particularly where equivalent accuracy to other AHRM devices was demonstrated. Strap fit, temperature, motion, wetness and tattoos interfered with recording accuracy.^74^ A usability study of a patch device for syncope found high acceptability, with minor issues relating to battery life, unclear indicators and occasional alerts from electrode contact loss.^52^

Implantable cardiac devices (ICDs and PPMs) were primarily therapeutic but also provided monitoring functions including battery status, sensing, lead impedance, rhythm detection and delivered therapy.^34 75 76^ There was high patient satisfaction with remote monitoring and no inferiority to in person follow up.^34 77^ As with ILRs, many patients reported wanting greater access to the information stored within their device.^38 75 76 78^ Issues mirrored ILRs but could be more complex and severe due to the long-term necessity for the device.

#### Reliability of evidence

Themes and subthemes emerged consistently across studies, contexts and populations, supporting relevance and robustness of findings. However, reliability is constrained by methodological limitations. The majority (95%) of studies were cohort or case–control designs (online supplemental file 1), making data susceptible to bias and confounding. Questionnaires were the predominant collection method, but only 32% were validated (n=10), and only two studies reported rationale for de novo questionnaire development. Limited patient involvement in questionnaire design suggests potential misalignment with patient priorities. Only 56% of studies were designed primarily to report patient factors. Nearly all studies were conducted in high-income countries (97.6%), limiting global generalisability.

## Discussion

**Figure 4:**
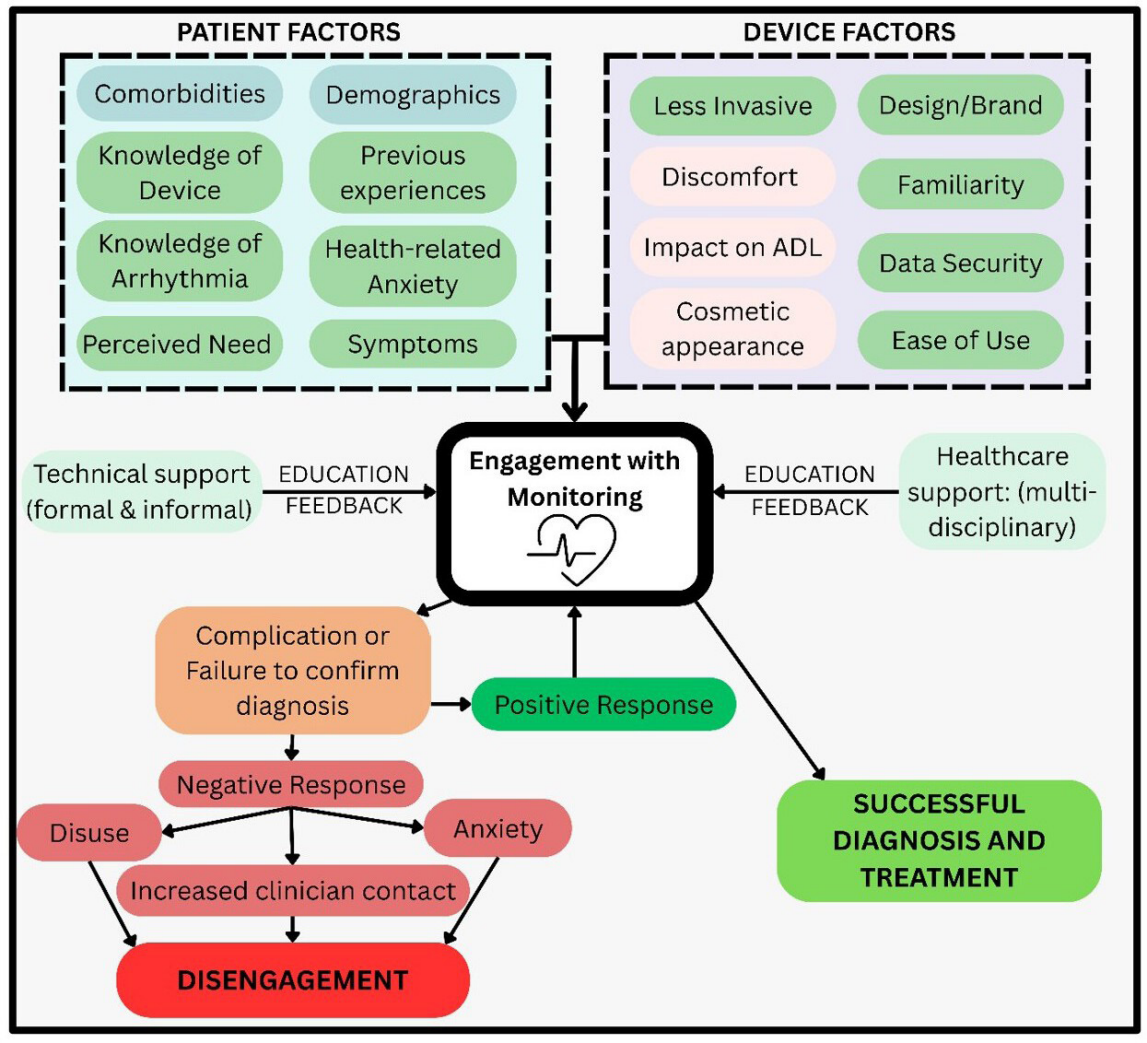
Visual representation of influences on engagement with ambulatory heart rhythm monitoring. ADL, activity of daily living.

This review identifies key patient-related and device-related factors influencing preference, experience and engagement with AHRM (figure 4). Well-designed, minimally invasive devices producing understandable, actionable feedback are favoured. Patient factors such as comorbidity, education and access to technology influence engagement. Wearable technologies, particularly smartwatches, increasingly appear to meet patient preferences.

Most studies were conducted in high-income countries in secondary and tertiary ambulatory care, focusing on older adults with cardiovascular comorbidities, especially AF. Younger populations, individuals with complex multimorbidity and those in resource-limited settings remain under-represented. This limited diversity constrains generalisability and highlights the need for more inclusive research to inform equitable arrhythmia surveillance. Studies that included more diverse populations (eg, learning difficulties, dialysis patients, living in rural settings) identified specific challenges requiring tailored consideration, as AHRM may increase burden of care in these groups.^29 30 37^ Further research is needed to characterise these issues, alongside learning from other remote monitoring fields; for example, evidence from rural telecardiology suggests that connectivity-related barriers may be mitigated through flexible transmission pathways and store-and-forward data uploads.^79^

Preferences of those with chronic conditions are complex and shaped by previous exposure to healthcare, stage of disease and aetiology.^80^ Wearable technology use is growing and could offer opportunities to integrate medical care with social functioning. However, they are predominantly direct-to-consumer, meaning information is often managed without medical supervision, possibly contributing to worry and increased healthcare usage. Frequent or automated alerts and increased self-monitoring may further amplify symptom awareness and health anxiety, particularly where findings are uncertain or lack clinical context.^44 73^ There is limited evidence on how these effects can be mitigated or prevented within current care models in literature. Given the increasing ownership of these devices and their capacity to generate complex health data that may influence patient experience and interaction with healthcare services, dedicated research in this area is needed. Education, technical support and feedback are key needs; how best to deliver these to maximise compliance and mitigate health anxiety requires further exploration in diverse populations.^81^,^83^ Most primary research consisted of cohort or case–control studies with heterogeneity in design, devices and outcomes. Questionnaires were rarely validated or developed with patient input. Nevertheless, although these methodological weaknesses are a limitation, the triangulation of quantitative and qualitative findings across studies adds strength to the conclusions reached.

Two dominant themes characterised experience and engagement: patient-related and device-related factors. **Patient factors** included comorbidity, knowledge, expectations and support. Older adults often demonstrated good adherence when adequately supported,^34^ consistent with other healthcare interactions such as medication adherence.^84^ Conversely, those with cognitive limitations or high treatment burden faced greater challenges. Although some populations may benefit from AHRM, delivering patient-centred monitoring remains challenging. A survey of follow-up modalities (n=1225) indicated that 71% favoured face-to-face review over video consultation, perceiving better care quality despite greater cost and travel.^85^ Patients valued communication with healthcare teams but often reported insufficient information regarding devices or results.^38 75 76 78^ Lack of premonitoring education was a recurrent barrier and unmet expectations sometimes generated anxiety.^38^ **Device factors** included invasiveness, ease of use, comfort and ability to integrate monitoring into daily life. Less invasive and discreet devices, such as patch ECGs and smartwatches, were generally preferred and caused fewer disruptions to sleep, exercise and work compared with Holter monitors.^50 58 70^ Differences between passive and active monitoring approaches may influence patient engagement and adherence; however, this distinction was not consistently reported across studies, limiting further analysis. Common problems included skin irritation, forgotten activation or charging, transmission failure and data security concerns.^36 64 71^

Non-invasive wearable technologies are developing rapidly and may offer capabilities comparable to ILRs, particularly when augmented by artificial intelligence.^7486^,^88^ Major technology companies are investing in biometric health devices driven by rising consumer demand.^89^ The global smartwatch market, currently valued at $44 billion, is expected to grow by 50% over 5 years.^90^ Companies are validating devices for medical purposes such as AHRM and facilitating integration of wearable data into electronic healthcare records—for example, Samsung recently acquired a data integration firm to facilitate direct data transfer for medical decision-making.^81 82 91^ Embedding heart rhythm monitoring into everyday accessories may reduce stigma by integrating monitoring into routine functions such as payments or messaging. Aligning this with realistic expectations is essential to avoid health anxiety and increased burden on patients and providers. Prospective research is required to clarify net impact and implementation challenges before large-scale adoption.

Key gaps persist. Evidence is largely drawn from high-income settings, leaving uncertainty regarding experience in resource-limited environments. Vulnerable populations and individuals with high treatment burden remain under-represented, and no studies addressed protected characteristics such as sex, ethnicity, religion or physical disability in relation to AHRM preferences. Future research should address these gaps. Meanwhile, clinical trials should prioritise easy-to-use, minimally invasive AHRM incorporating clear feedback and support tailored to patient context and capacity for self-management.

Although this review focuses on patient perspectives, AHRM decision-making involves other stakeholders. A minority of clinicians diverge from guidelines, often underusing longer-term monitoring such as ILRs.^92 93^ The role of wearable devices in arrhythmia management is increasingly recognised in guidelines;^94^ however, studies examining clinician attitudes identify several potential implementation challenges. The DAS-CAM III study (n=500) found clinicians preferred ECG over photoplethysmography (PPG)-based devices for AF investigation and frequently ordered confirmatory ECG following PPG alerts.^95^ The wEHRAbles 2 survey (n=539) reported broad acceptance of wearables for arrhythmia diagnosis but less confidence in using them for anticoagulation decisions.^96^ Although evidence supporting PPG-based AF detection is growing, it remains less well validated than ECG and is currently recommended for screening only; confirmatory ECG is therefore required before clinical decision-making in current guidelines.^94^ However, uptake of these technologies may also be influenced by non-clinical factors that limit clinician confidence, potentially leading to unnecessary investigations and hindering adoption. Clinician behaviour is complex and often resistant to practice change;^97^ addressing this is essential for translation of research into practice.

### Limitations

Searches were conducted across relevant databases, with terms agreed to group commonalities. This may have missed some papers or led to small differences in grouping. Most studies underwent single screening; however, test samples that were double screened showed concordance and any uncertainties were resolved through team discussion. Conference abstracts and non-English language papers were excluded, though their impact is likely small. We did not conduct a risk of bias assessment as the aim of this scoping review was to map evidence rather than produce a definitive synthesis, but considered study design when assessing evidence limitations—this is in-keeping with best practice methodology.^21^

## Conclusion

AHRM is shaped by patient-related and device-related factors, influencing uptake and outcomes. Current evidence highlights a preference for minimally invasive, user-friendly technologies accompanied by clear feedback and support. Future research must address evidence gaps in diverse populations and low-resource settings to inform equitable, patient-centred arrhythmia care.

## Supplementary material

10.1136/bmjopen-2025-110631 online supplemental file 1

## Data Availability

All data relevant to the study are included in the article or uploaded as supplementary information.
