# Supplementary material for: Understanding patient preferences, experiences and engagement with ambulatory heart rhythm monitoring: a scoping review
Source: BMJ Open. 2026 May 8;16(5):e110631. doi: 10.1136/bmjopen-2025-110631 (PMC13157736; doi:10.1136/bmjopen-2025-110631)
Supplement: online supplemental file 1 [file bmjopen-16-5-s001.pdf]

Supplementary Data Table 1 – Comparison of commonly used devices for ambulatory heart rhythm monitoring (AHRM).

Activities of Daily Living (AoDL); Electrocardiogram (ECG); Food and Drug Administration (FDA); Atrial Fibrillation (AF); Photoplethysmography (PPG).

	**Holter Monitors**	**Patch Devices**	**Event Recorders**	**Implantable Loop Recorders**	**External Loop Recorder / Mobile Cardiac Telemetry**	**Wearable Devices**	**Smartwatches**	**Implantable cardiac devices (pacemakers / implantable cardiac defibrillators)**
**First used / FDA approved**	1960s [S1]	2009 [S2]	1980s	2001 [S3]	2002 [S4]	2000s	2018 [S5]	1979 [S6]
**Device Design**	Box-like device worn on a belt or shoulder strap with electrode sensors attached the chest	Single slim, flexible, adhesive, resembling a large sticker, placed on the chest	Small, rectangular handheld device with button electrode sensors used to record ECG	Small, rectangular device implanted under the skin on left chest wall.	Compact, box-like device attached to skin or carried, with electrode sensors attached to the chest.	Range of forms with sensors integrated into chest straps, vests, finger rings or glove device	Digital watch, worn on wrist, with flat metal sensors integrated into the watch back and bezel crown	Compact, battery-powered device placed under the skin with leads that deliver electrical pulses directly to the heart
**Electrode stickers used?**	Multiple small adhesive patches, connected by wires to device	Large adhesive patch with electrode embedded	No	No	Multiple small adhesive patches, connected directly or by wires to device	Device dependent. If yes, electrodes attached with multiple small adhesive patches.	No	No
**Number of leads**	5 to 7	1	1 to 6	1	2 to 6	1 to 6	1	1
**Length of use**	24 - 72 hours, up to 14 days [51]	Up to 14 days [58, S7]	Weeks – Months [S8]	Up to 4 years [S9]	Up to 30 days [S10]	Varies, based on device	No specified limit	Device often placed indefinitely

**How is data collected?**	Continuous	Continuous	Intermittent	Intermittent	Intermittent	Varies, based on device	PPG = continuous; ECG = intermittent	Continuous
**How is ECG recorded?**	ECG data is continuously collected and is automatic.	ECG data is continuously collected and is automatic.	Solely patient activated. Patient holds device in two hands, sometimes against a part of the body and presses buttons to record	Based on pre-set criteria or when patient activated, by pressing button on device	Triggered by abnormal cardiac rhythm or patient activated, by pressing button on device	Varies, based on device.	PPG data is continuous; single-lead ECG is patient-activated by app. To record a finger is placed on the watch bezel / crown	Built-in sensing electrodes on the device's leads collect ECG data.
**Marking symptoms**	Symptom diary	Button on device	Symptom initiated	Remote recording with device	Through monitor provided with device	Varies, based on device	Either through app or at time of single-lead ECG recording	Symptom diary or mark events via an associated app or device
**Placement**	Requires healthcare attendance	Self-applied	Self-applied	Invasive procedure	Self-applied	Self-applied	Self-applied	Invasive procedure
**Arrhythmia burden information**	Yes	Yes	No	Yes	Yes	Varies, based on device	Yes	Yes
**Approximate yield of diagnosis**	Symptoms = 5-39% [S11] Syncope = 6-22% [S12, S13]	Symptoms = 62.2% [S14] Syncope = 10.5% [S15]	Symptoms = 52.8-76.4% [S16, S17]	Symptoms = 60-73% [S18, S19] Syncope = Up to 80% (4 year follow-up) [S20]	Symptoms = 66-83% [S21] Syncope = 25-56% [S12]	Yet to be validated for investigation of symptoms / syncope	Yet to be validated for investigation of symptoms / syncope	Not validated for investigation of symptoms / syncope
**Sensitivity / Specificity for AF detection**	Sensitivity = 96.3% Specificity = 96.8% [S22]	Sensitivity = 96% Specificity = 99% (S23]	Sensitivity = 80-100% Specificity =88-99% [S24]	Sensitivity = 79-95% Specificity = 66-100%[S25, S26]	Sensitivity = 84% Specificity = 98% [S27]	Sensitivity = 83-99%, Specificity = 95.6-99% [S28]	Sensitivity = 96.8% Specificity = 81.1-99.6% [S29]	Sensitivity = 88.6-98.3% Specificity = 95.4–100.0% [S30]
**Data transfer**	Device returned for analysis	Device returned for analysis	Uploads remotely via mobile device /	Uploads remotely via mobile device /	Uploads remotely via mobile device /	Uploads remotely via mobile device /	Uploads remotely via mobile device /	Uploads remotely via mobile device /

			home equipment	home equipment	home equipment	home equipment	home equipment	home equipment or on in-person interrogation of device
**Specific issues**	- Skin irritation [71]<br>- Cannot get device wet [50]<br>- High impact on AoDL [57,58]<br>- Motion artefact / electrode detachment [S31, S32]<br>- Poor compliance, with longer use (>72 hours)	- Skin irritation [S33]<br>- Higher relative cost as not reusable [S34]	- Rely on patient to activate to record, may miss asymptomatic arrhythmia [S35, S36]<br>- Limited information on duration or burden of arrhythmia [35]	- Procedure-related complications i.e. pain, bleeding, infection [40,48]<br>- Requires repeat invasive procedure to remove<br>- Rare reports of device migration (requiring removal) [S37 – S39]	- Skin irritation [S40]<br>- Cannot get device wet [S40]<br>- Need to replace electrodes often [S40]<br>- High cost and resource demand [S41]<br>- No continuous recording capabilities	- Varies, based on device	- Rely on patient to activate ECG to record [S42, S43]<br>- High variation of ECG quality [S44]<br>- Privacy and data concerns [S45]<br>- Commercially available with limited evidence base for use	- Procedure-related complications i.e. pain, bleeding, infection [S46]<br>- Periodic replacement of batteries [S46]<br>- If issues with device, requires repeat procedure to remove [S46]<br>- Inappropriate shocks [S46]

Supplementary Data Table 2 – Search strategy used for PubMed

Each part of the research question was split into concepts to identify keywords. These were searched within the Medical Subject Headings (MeSH) section of PubMed to identify relevant indexed terms to include. A combination of both keywords and MeSH terms were used in the final search. The same approach was used in all databases and indexed terms, where applicable, were used.

<u>Concept 1: Patient choice</u>

Keywords

“patient choice*”[tw] OR “patient selection*”[tw] OR “patient decision*”[tw] OR “patient decision-making” [tw] OR “patient preference*”[tw] OR “patient behaviour*”[tw]

Medical Subject Headings (MeSH)

"Choice Behavior"[Mesh] OR "Public Opinion"[Mesh] OR "Patient Participation"[Mesh] OR "Decision Making"[Mesh] OR "Clinical Decision-Making"[Mesh] OR "Decision Making, Shared"[Mesh] OR "Patient Preference"[Mesh] OR "Treatment Adherence and Compliance"[Mesh] OR "Attitude to Health"[Mesh] OR "Health Behavior"[Mesh]

Combined

“patient choice*”[tw] OR “patient selection*”[tw] OR “patient decision*”[tw] OR “patient decision-making” [tw] OR “patient preference*”[tw] OR “patient behaviour*”[tw] OR "Choice Behavior"[Mesh] OR "Public Opinion"[Mesh] OR "Patient Participation"[Mesh] OR "Decision Making"[Mesh] OR "Clinical Decision-Making"[Mesh] OR "Decision Making, Shared"[Mesh] OR "Patient Preference"[Mesh] OR "Treatment Adherence and Compliance"[Mesh] OR "Attitude to Health"[Mesh] OR "Health Behavior"[Mesh]

<u>Concept 2 : Patient engagement</u>

Keywords

“patient engag*”[tw] OR “patient encourag*”[tw] OR “patient involv*” OR “patient relation*”[tw]

Medical Subject Headings (MeSH)

"Patient Participation"[Mesh] OR "Physician-Patient Relations"[Mesh] OR "Professional-Patient Relations"[Mesh]

Combined

“patient engag*”[tw] OR “patient encourag*”[tw] OR “patient involv*” OR “patient relation*”[tw] OR "Patient Participation"[Mesh] OR "Physician-Patient Relations"[Mesh] OR "Professional-Patient Relations"[Mesh]

Concept 3 : Ambulatory care

Keywords

“outpatient*”[tw] OR “clinic”[tw] OR “ambulatory care” [tw] OR “ambulatory monitor*”[tw] OR mHealth[tw] OR “wearable device*”[tw] OR smartwatch*[tw] OR “smartphone monitor*”[tw] OR “wearable device*” [tw]

Medical Subject Headings (MeSH)

"Outpatients"[Mesh] OR "Outpatient Clinics, Hospital"[Mesh] OR "Ambulatory Care"[Mesh] OR "Ambulatory Care Facilities"[Mesh] OR "Monitoring, Ambulatory"[Mesh]OR "Telemedicine"[Mesh] OR "Delivery of Health Care"[Mesh] OR "Wearable Electronic Devices"[Mesh]

Combined

“outpatient*”[tw] OR “clinic”[tw] OR “ambulatory care” [tw] OR “ambulatory monitor*”[tw] OR mHealth[tw] OR “wearable device*”[tw] OR smartwatch*[tw] OR “smartphone monitor*”[tw] OR “wearable device*” [tw] OR "Outpatients"[Mesh] OR "Outpatient Clinics, Hospital"[Mesh] OR "Ambulatory Care"[Mesh] OR "Ambulatory Care Facilities"[Mesh] OR "Monitoring, Ambulatory"[Mesh]OR "Telemedicine"[Mesh] OR "Delivery of Health Care"[Mesh] OR "Wearable Electronic Devices"[Mesh]

Concept 4 : Electrocardiography monitoring

Keywords

“cardiac monitor*”[tw] OR “heart monitor*”[tw] OR “arrhythmia monitor*”[tw] OR “electrocardiography monitor*”[tw]

Medical Subject Headings (MeSH)

"Electrocardiography, Ambulatory"[Mesh]

Combined

“cardiac monitor*”[tw] OR “heart monitor*”[tw] OR “arrhythmia monitor*”[tw] OR “electrocardiography monitor*”[tw] OR "Electrocardiography, Ambulatory"[Mesh]

Search 1 = Concept 1 AND Concept 3 AND Concept 4

Search 2 = Concept 2 AND Concept 3 AND Concept 4

Supplementary Data Table 3 – Summary of all primary research articles included within the study.

External Loop Recorders (ELRs); Implantable Cardioverter Defibrillator (ICD); Implantable Loop Recorder (ILR); Levels of Evidence (LoE); Mobile Cardiac Telemetry (MCT); Oxford Centre for Evidence-Based Medicine (OCEBM); Permanent Pacemaker (PPM)

Author	Year	Country	Sample (n)	Holter	Patch Device	Event Recorder	ILR	ELRs / MCTs	Wearable Devices	Smart watches	ICD	PPM	Research methodology	Qualitative Component	Funding	LoE (OCEBM)
Scherr et al	2008	New Zealand	18	X		X							Quantitative	-	Government	2b
Fensli et al	2010	Norway	36		X								Multiple	Semi-structured interviews	Not stated	2b
Ricci et al	2010	Italy	119								X	X	Quantitative	-	Not stated	3b
Furukawa et al	2011	Italy	47				X						Quantitative	-	Not stated	2b
Roten et al	2012	Switzerland	100	X				X					Quantitative	-	Industry	3b
Ackermans et al	2012	USA	62		X								Quantitative	-	Not stated	1b
Carter et al	2012	USA	31						X				Quantitative	-	Academic Institution	2b
Petersen et al	2012	Denmark	358								X		Multiple	Free text box	Not stated	3b
Schlingloff et al	2013	Germany	70				X						Quantitative	-	Industry	2b
Prescher et al	2013	Germany	228			X							Multiple	Free text box	Industry / Government	3b
Tu et al	2014	Australia	20	X									Quantitative	-	Not stated	2b
Barrett et al	2014	USA	146	X	X								Quantitative	-	Not stated	2b
Costa et al	2015	Portugal	11						X				Quantitative	-	Government	2b
Yong et al	2016	USA	NA	X	X								Quantitative	-	Not stated	3b
Lowres et al	2016	Australia	44			X							Mixed	Semi-structured interviews	Industry / Academic Institution	3b
El Hage et al	2017	USA	46				X						Multiple	Free text box	Academic Institution	3b
Smith et al	2017	New Zealand	50	X	X								Quantitative	-	Not stated	2b
Timmermans et al	2017	-	-								X		Review (quantitative and qualitative)	-	-	-
Reading et al	2018	USA	13			X							Qualitative	Focus group	Academic Institution	2b
Narasimha et al	2018	USA	33			X							Quantitative	-	Not stated	3b
Bisignani et al	2018	Italy	55				X						Quantitative	-	Industry	2b
Reinsch et al	2018	USA	30				X						Quantitative	-	Not stated	2b

Lee et al	2018	South Korea	224	X	X	X	X						Mixed	Semi-structured interviews	Not stated	3b
Majid et al	2018	-	-	X	X	X	X	X					Review (qualitative)	-	-	-
Karaoğuz et al	2019	Turkey	109		X								Quantitative	Free text box	Not stated	2b
Eysenck et al	2019	UK	21	X	X							X	Quantitative	-	Not stated	2b
Patel et al	2019	USA	344								X	X	Multiple	Free text box	Not stated	3b
Versteeg et al	2019	France, Germany, Spain, Switzerland, Netherlands	595								X		Quantitative	-	Industry	1b
Villani et al	2019										X	X				
Lumikari et al	2020	Finland	15						X				Multiple	Free text box	Government	2b
Fredriksson et al	2020	Sweden	269	X	X								Quantitative	-	Industry	2b
Ventura et al	2020	USA	12				X						Qualitative	Semi-structured interviews	Not stated	3b
Sears et al	2020	USA	51			X					X		Quantitative	-	Academic Institution / Industry	2b
Masterson Creber et al	2021	USA	105			X							Quantitative	-	Academic Institution	2b
Nuvvula et al	2021	USA	1222						X	X			Quantitative	-	Industry	2b
Lee et al	2021	USA	128			X							Mixed	Free text box	Academic Institution	3b
Xu et al	2021	USA	12				X						Qualitative	Focus group	Academic Institution	3b
Altinsoy et al	2021	USA, Germany, Japan, UK,	215	X	X		X	X					Quantitative	-	Industry	3b
Ventura et al	2021	USA	12				X						Qualitative	Semi-structured interviews	Not stated	3b
Birs et al	2022	USA	28				X						Quantitative	-	Industry / Academic Institution	3b
Pearson et al	2022	-	-			X							Review (quantitative)	-	-	-
Otabil et al	2023	USA	120		X								Quantitative	-	Academic Institution	2b
Mannhart et al	2023	Switzerland	201							X			Multiple	Free text box	Industry	2b
Steinhauer et al.	2024	Switzerland	321				X						Multiple	Free text box	Not stated	2b
Patrick et al	2024	USA	95		X								Quantitative	-	Not stated	3b
Junarta et al	2024	USA	184		X								Quantitative	-	Not stated	2b

Vyas et al	2024	-	-						X	X			Review (quantitative and qualitative)	-	-	-
Hassani et al	2024	-	-	X	X	X	X	X		X			Review (quantitative and qualitative)	-	-	-
Mathew et al.	2024	USA	104		X					X			Quantitative	-	Government	2b
Rosman et al.	2024	USA	172						X	X			Quantitative	-	Government	2b
Pearsons et al.	2025	UK	20		X								Qualitative	Semi-structured interviews	Government	3b
Dzikowicz et al	2025	USA	256		X								Quantitative	-	Not stated	2b
Zeitler et al.	2025	USA	23					X			X	X	Qualitative	Semi-structured interviews	Not stated	3b
Karregat et al.	2025	Netherlands	18	X						X			Qualitative	Semi-structured interviews	Government / Academic Institution	3b
Runge et al.	2025	Switzerland	9			X			X				Quantitative	-	Government / Academic Institution	2b
Clarysse et al	2026	Belgium	753							X			Quantitative	-	Academic Institution	3b

Supplementary Data Table 4 – List of validated questionnaires used to assess patient related factors for AHRM

Atrial Fibrillation Effect on Quality of Life (n=3)
University of Toronto Atrial Fibrillation Severity Scale (n=2)
Cardiac Anxiety Questionnaire (n=2)
Generalized Anxiety Disorder-7 scale (n=2)
Physical Component Summary and Mental Component Summary of the 12-Item validated Short Form Survey (n=1)
zMobile Application Rating Scale (n=1)
Self-efficacy for Appropriate Medication Use Scale (n=1)
The Control-Attitudes Scale-Revised (n=1)
Systems Usability Scale (n=1)
Atrial Fibrillation Knowledge Scale (n=1)
Florida Shock Anxiety Scale (n=1)
Florida Patient Acceptance Survey (n=1)
Patient-Reported Outcomes Measurement Information System (n=1)
12-Item Short Form Health Survey (n=1)
Consumer Health Activation Index (n=1)
UTAUT2 (Extended Unified Theory of Acceptance and Use of Technology) (n=1)

Supplementary Data Table 5 – Contribution of individual papers to specific sub-sections of the Results

Author	Year	Patient or device factors	Which patient factors did these studies contribute to?	Which device factors did this study contribute to?
Scherr et al	2008	Both	• Experience and Preferences	• General Factors<br>• Specific Factors
Fensli et al	2010	Both	• Education and Expectations<br>• Impact of Monitoring on Activities of Daily Living (ADL) and Healthcare Interaction	• Specific Factors
Ricci et al	2010	Patient	• Clinical and Demographic Factors<br>• Impact of Monitoring on Activities of Daily Living (ADL) and Healthcare Interaction	
Furukawa et al	2011	Both	• Experience and Preferences<br>• Impact of Monitoring on Activities of Daily Living (ADL) and Healthcare Interaction	• General Factors
Roten et al	2012	Device		• General Factors<br>• Specific Factors
Ackermans et al	2012	Both	• Impact of Monitoring on Activities of Daily Living (ADL) and Healthcare Interaction	• General Factors<br>• Specific Factors
Carter et al	2012	Both	• Experience and Preferences<br>• Impact of Monitoring on Activities of Daily Living (ADL) and Healthcare Interaction	• General Factors
Petersen et al	2012	Both	• Experience and Preferences<br>• Impact of Monitoring on Activities of Daily Living (ADL) and Healthcare Interaction	• Specific Factors
Schlingloff et al	2013	Both	• Clinical and Demographic Factors	• General Factors<br>• Specific Factors
Prescher et al	2013	Both	• Education and Expectations<br>• Experience and Preferences<br>• Impact of Monitoring on Activities of Daily Living (ADL) and Healthcare Interaction	• General Factors
Tu et al	2014	Device		• General Factors
Barrett et al	2014	Both	• Experience and Preferences<br>• Impact of Monitoring on Activities of Daily Living (ADL) and Healthcare Interaction	• Specific Factors
Costa et al	2015	Both	• Experience and Preferences<br>• Impact of Monitoring on Activities of Daily Living (ADL) and Healthcare Interaction	• Specific Factors
Yong et al	2016	Patient	• Impact of Monitoring on Activities of Daily Living (ADL) and Healthcare Interaction	
Lowres et al	2016	Both	• Clinical and Demographic Factors<br>• Education and Expectations<br>• Experience and Preferences<br>• Impact of Monitoring on Activities of Daily Living (ADL) and Healthcare Interaction	• Specific Factors
El Hage et al	2017	Patient	• Clinical and Demographic Factors	
Smith et al	2017	Both	• Experience and Preferences<br>• Impact of Monitoring on Activities of Daily Living (ADL) and Healthcare Interaction	• General Factors<br>• Specific Factors
Timmermans et al	2017	Both	• Clinical and Demographic Factors<br>• Impact of Monitoring on Activities of Daily Living (ADL) and Healthcare Interaction	• Specific Factors
Reading et al	2018	Both	• Clinical and Demographic Factors<br>• Education and Expectations<br>• Experience and Preferences<br>• Impact of Monitoring on Activities of Daily Living (ADL) and Healthcare Interaction	• Specific Factors
Narasimha et al	2018	Both	• Experience and Preferences<br>• Impact of Monitoring on Activities of Daily Living (ADL) and Healthcare Interaction	• General Factors
Bisignani et al	2018	Both	• Experience and Preferences	• General Factors<br>• Specific Factors

Reinsch et al	2018	Both	• Education and Expectations<br>• Experience and Preferences<br>• Impact of Monitoring on Activities of Daily Living (ADL) and Healthcare Interaction	• General Factors<br>• Specific Factors
Lee et al	2018	Both	• Education and Expectations<br>• Experience and Preferences<br>• Impact of Monitoring on Activities of Daily Living (ADL) and Healthcare Interaction	• General Factors
Majid et al	2018	Patient	• Education and Expectations<br>• Experience and Preferences<br>• Impact of Monitoring on Activities of Daily Living (ADL) and Healthcare Interaction	
Karaoğuz et al	2019	Both	• Experience and Preferences<br>• Impact of Monitoring on Activities of Daily Living (ADL) and Healthcare Interaction	• General Factors<br>• Specific Factors
Eysenck et al	2019	Both	• Impact of Monitoring on Activities of Daily Living (ADL) and Healthcare Interaction	• General Factors<br>• Specific Factors
Patel et al	2019	Both	• Education and Expectations<br>• Impact of Monitoring on Activities of Daily Living (ADL) and Healthcare Interaction	• Specific Factors
Versteeg et al	2019	Device		• Specific Factors
Villani et al	2019	Both	• Impact of Monitoring on Activities of Daily Living (ADL) and Healthcare Interaction	• Specific Factors
Lumikari et al	2020	Both	• Experience and Preferences<br>• Impact of Monitoring on Activities of Daily Living (ADL) and Healthcare Interaction	• Specific Factors
Fredriksson et al	2020	Both	• Education and Expectations<br>• Experience and Preferences<br>• Impact of Monitoring on Activities of Daily Living (ADL) and Healthcare Interaction	• General Factors<br>• Specific Factors
Ventura et al	2020	Patient	• Education and Expectations<br>• Experience and Preferences<br>• Impact of Monitoring on Activities of Daily Living (ADL) and Healthcare Interaction	
Sears et al	2020	Device		• Specific Factors
Masterson Creber et al	2021	Patient	• Clinical and Demographic Factors	
Nuvvula et al	2021	Patient	• Experience and Preferences<br>• Impact of Monitoring on Activities of Daily Living (ADL) and Healthcare Interaction	
Lee et al	2021	Patient	• Clinical and Demographic Factors	
Xu et al	2021	Both	• Clinical and Demographic Factors<br>• Education and Expectations<br>• Experience and Preferences<br>• Impact of Monitoring on Activities of Daily Living (ADL) and Healthcare Interaction	• Specific Factors
Altinsoy et al	2021	Patient	• Education and Expectations<br>• Experience and Preferences	
Ventura et al	2021	Both	• Education and Expectations<br>• Experience and Preferences<br>• Impact of Monitoring on Activities of Daily Living (ADL) and Healthcare Interaction	• Specific Factors
Birs et al	2022	Device		• General Factors<br>• Specific Factors
Pearson et al	2022	Both	• Clinical and Demographic Factors<br>• Experience and Preferences<br>• Impact of Monitoring on Activities of Daily Living (ADL) and Healthcare Interaction	• General Factors
Otabil et al	2023	Patient	• Experience and Preferences	
Mannhart et al	2023	Both	• Experience and Preferences	• Specific Factors

Steinhauer et al.	2024	Both	• Education and Expectations<br>• Experience and Preferences<br>• Impact of Monitoring on Activities of Daily Living (ADL) and Healthcare Interaction	• General Factors<br>• Specific Factors
Patrick et al	2024	Patient	• Clinical and Demographic Factors	
Junarta et al	2024	Patient	• Clinical and Demographic Factors<br>• Impact of Monitoring on Activities of Daily Living (ADL) and Healthcare Interaction	
Vyas et al	2024	Both	• Experience and Preferences<br>• Impact of Monitoring on Activities of Daily Living (ADL) and Healthcare Interaction	• Specific Factors
Hassani et al	2024	Both	• Experience and Preferences	• Specific Factors
Mathew et al.	2024	Patient	• Clinical and Demographic Factors	
Rosman et al.	2024	Patient	• Experience and Preferences<br>• Impact of Monitoring on Activities of Daily Living (ADL) and Healthcare Interaction	
Pearsons et al.	2025	Both	• Experience and Preferences<br>• Impact of Monitoring on Activities of Daily Living (ADL) and Healthcare Interaction	• Specific Factors
Dzikowicz et al	2025	Patient	• Clinical and Demographic Factors	
Zeitler et al.	2025	Device	• Education and Expectations<br>• Impact of Monitoring on Activities of Daily Living (ADL) and Healthcare Interaction	
Karregat et al.	2025	Both	• Clinical and Demographic Factors<br>• Education and Expectations<br>• Experience and Preferences<br>• Impact of Monitoring on Activities of Daily Living (ADL) and Healthcare Interaction	• Specific Factors
Runge et al.	2025	Patient	• Experience and Preferences	
Clarysse et al	2026	Patient	• Experience and Preferences	

Supplementary References

S1. Mar BD. The history of clinical Holter monitoring. *Ann Noninvasive Electrocardiol.* 2005;10(2):226–230.

S2. US Food and Drug Administration. 510(k) Premarket Notification [Internet]. [cited 2025 Jan 27]. Available from: https://www.accessdata.fda.gov/scripts/cdrh/cfdocs/cfpmn/pmn.cfm?ID=K090363

S3. US Food and Drug Administration. Premarket Approval (PMA) [Internet]. [cited 2025 Jan 27]. Available from: https://www.accessdata.fda.gov/scripts/cdrh/cfdocs/cfpma/pma.cfm?id=P890003S062

S4. Zweibel S, Trelfa M. The use of mobile cardiac telemetry to improve diagnostic accuracy and enable more efficient patient care [Internet]. 2012 [cited 2025 Jan 27]. Available from: https://www.uscjournal.com/articles/use-mobile-cardiac-telemetry-improve-diagnostic-accuracy-and-enable-more-efficient-patient

S5. US Food and Drug Administration. De novo classification request for ECG app [Internet]. [cited 2025 Jan 27]. Available from: https://www.accessdata.fda.gov/cdrh_docs/reviews/DEN180042.pdf

S6. Rome BN, Kramer DB, Kesselheim AS. FDA approval of cardiac implantable electronic devices via original and supplement premarket approval pathways, 1979–2012. *JAMA.* 2014;311(4):385–391.

S7. Yenikomshian M, Jarvis J, Patton C, Yee C, Mortimer R, Birnbaum H, et al. Cardiac arrhythmia detection outcomes among patients monitored with the Zio patch system: a systematic literature review. *Curr Med Res Opin.* 2019;35(10):1659–1670.

S8. Wegner FK, Kochhäuser S, Ellermann C, Lange PS, Frommeyer G, Leitz P, et al. Prospective blinded evaluation of the smartphone-based AliveCor Kardia ECG monitor for atrial fibrillation detection: the PEAK-AF study. *Eur J Intern Med.* 2020;73:72–75.

S9. Bisignani A, De Bonis S, Mancuso L, Ceravolo G, Bisignani G. Implantable loop recorder in clinical practice. *J Arrhythm.* 2019;35(1):25–32.

S10. Olson JA, Fouts AM, Padanilam BJ, Prystowsky EN. Utility of mobile cardiac outpatient telemetry for the diagnosis of palpitations, presyncope, syncope, and the assessment of therapy efficacy. *J Cardiovasc Electrophysiol.* 2007;18(5):473–477.

S11. Weinstock C, Wagner H, Snuckel M, Katz M. Evidence-based approach to palpitations. *Med Clin North Am.* 2021;105(1):93–106.

S12. Ruwald MH, Zareba W. ECG monitoring in syncope. *Prog Cardiovasc Dis.* 2013;56(2):203–210.

S13. Burkowitz J, Merzenich C, Grassme K, Brüggenjürgen B. Insertable cardiac monitors in the diagnosis of syncope and the detection of atrial fibrillation: a systematic review and meta-analysis. *Eur J Prev Cardiol.* 2016;23(12):1261–1273.

S14. Lobodzinski SS. ECG patch monitors for assessment of cardiac rhythm abnormalities. *Prog Cardiovasc Dis.* 2013;56(2):224–229.

S15. Reed MJ, Grubb NR, Lang CC, Gray AJ, Simpson K, MacRaild A, et al. Diagnostic yield of an ambulatory patch monitor in patients with unexplained syncope after initial evaluation in the emergency department: the PATCH-ED study. *Emerg Med J.* 2018;35(8):477–485.

S16. Torres Gonzalez N, Alvarez-Acosta L, Valdivia-Miranda D, Barreto-Caceres V, Iriarte-Plasencia A, Cappellesso P, et al. Diagnostic yield of a portable electrocardiographic device versus 24-hour conventional Holter monitoring in the diagnosis of arrhythmic events. *Europace.* 2022;24(Suppl 1):euac053.570.

S17. British Journal of Cardiology. Diagnostic utility of real-time smartphone ECG in the initial investigation of palpitations [Internet]. [cited 2025 Jan 30]. Available from: https://bjcardio.co.uk/2018/03/diagnostic-utility-of-real-time-smartphone-ecg-in-the-initial-investigation-of-palpitations/

S18. Smith A, Perdue M, Vojnika J, Frisch DR, Pavri BB. The diagnostic yield of implantable loop recorders stratified by indication: real-world use in a large academic hospital. *J Interv Card Electrophysiol.* 2021;61(2):303–311.

S19. Giada F, Gulizia M, Francese M, Croci F, Santangelo L, Santomauro M, et al. Recurrent unexplained palpitations (RUP) study. *J Am Coll Cardiol.* 2007;49(19):1951–1956.

S20. Furukawa T, Maggi R, Bertolone C, Fontana D, Brignole M. Additional diagnostic value of very prolonged observation by implantable loop recorder in patients with unexplained syncope. *J Cardiovasc Electrophysiol.* 2012;23(1):67–71.

S21. Zimetbaum P, Josephson ME. Evaluation of patients with palpitations. *N Engl J Med.* 1998;338(19):1369–1373.

S22. Jiang K, Huang C, Ye SM, Chen H. High accuracy in automatic detection of atrial fibrillation for Holter monitoring. *J Zhejiang Univ Sci B.* 2012;13(9):751–759.

S23. Noseworthy PA, Kaufman ES, Chen LY, Chung MK, Elkind MSV, Joglar JA, et al. Subclinical and device-detected atrial fibrillation: a scientific statement from the American Heart Association. *Circulation.* 2019;140(25):e944–e963.

S24. Zungsontiporn N, Link MS. Newer technologies for detection of atrial fibrillation. *BMJ.* 2018;363:k3946.

S25. Podd SJ, Sugihara C, Furniss SS, Sulke N. Are implantable cardiac monitors the gold standard for atrial fibrillation detection? *Europace.* 2016;18(7):1000–1005.

S26. Kusiak A, Jastrzębski M, Bednarski A, Kułakowski P, Piotrowski R, Koźluk E, et al. Diagnostic value of implantable loop recorder in patients undergoing cryoballoon ablation of atrial fibrillation. *Ann Noninvasive Electrocardiol.* 2020;25(4):e12733.

S27. Sejr MH, May O, Damgaard D, Sandal BF, Nielsen JC. External continuous ECG versus loop recording for atrial fibrillation detection in patients who had a stroke. *Heart.* 2019;105(11):848–854.

S28. Dahiya ES, Kalra AM, Lowe A, Anand G. Wearable technology for monitoring electrocardiograms (ECGs) in adults: a scoping review. *Sensors (Basel).* 2024;24(4):1318.

S29. Belani S, Wahood W, Hardigan P, Placzek AN, Ely S. Accuracy of detecting atrial fibrillation: a systematic review and meta-analysis of wrist-worn wearable technology. *Cureus.* 2021;13(12).

S30. Baalman SWE, Mittal S, Boersma LVA, Perschbacher D, Brisben AJ, Mahajan D, et al. Real-world performance of the atrial fibrillation monitor in patients with a subcutaneous ICD. *Pacing Clin Electrophysiol.* 2020;43(12):1467–1475.

S31. de Voogt WG, van Hemel NM, van de Bos AA, Koïstinen J, Fast JH. Verification of pacemaker automatic mode switching for the detection of atrial fibrillation and atrial tachycardia with Holter recording. *Europace.* 2006;8(11):950–961.

S32. Lee J, McManus DD, Merchant S, Chon KH. Automatic motion and noise artifact detection in Holter ECG data using empirical mode decomposition and statistical approaches. *IEEE Trans Biomed Eng.* 2012;59(6):1499–1506.

S33. Turakhia MP, Hoang DD, Zimetbaum P, Miller JD, Froelicher VF, Kumar UN, et al. Diagnostic utility of a novel leadless arrhythmia monitoring device. *Am J Cardiol.* 2013;112(4):520–524.

S34. Reynolds MR, Passman R, Swindle J, Mohammadi I, Wright B, Boyle K, et al. Comparative effectiveness and healthcare utilization for ambulatory cardiac monitoring strategies in Medicare beneficiaries. *Am Heart J.* 2024;269:25–34.

S35. Goldenthal IL, Sciacca RR, Riga T, Bakken S, Baumeister M, Biviano AB, et al. Recurrent atrial fibrillation/flutter detection after ablation or cardioversion using the AliveCor KardiaMobile device: iHEART results. *J Cardiovasc Electrophysiol.* 2019;30(11):2220–2228.

S36. Halcox JPJ, Wareham K, Cardew A, Gilmore M, Barry JP, Phillips C, et al. Assessment of remote heart rhythm sampling using the AliveCor heart monitor to screen for atrial fibrillation. *Circulation.* 2017;136(19):1784–1794.

S37. Rahkovich M, Laish-Farkash A. Migration of a new generation implantable loop recorder: a case report. *Eur Heart J Case Rep.* 2021;5(2):ytab043.

S38. Russo V, Sica G, Mauriello A, Casazza D, Rago A. Migration of long-sensing vector implantable loop recorder unmasked by remote monitoring in patient with unexplained syncope. *J Cardiol Cases.* 2024;30(2):51–54.

S39. Squillace M, Aurino L, Makmur G, Durante A. Pleural cavity migration of an implantable loop recorder. *IHJ Cardiovasc Case Rep.* 2023;7(3):83–84.

S40. Steinberg JS, Varma N, Cygankiewicz I, Aziz P, Balsam P, Baranchuk A, et al. 2017 ISHNE-HRS expert consensus statement on ambulatory ECG and external cardiac monitoring/telemetry. *Heart Rhythm.* 2017;14(7):e55–e96.

S41. Zimetbaum P, Goldman A. Ambulatory arrhythmia monitoring: choosing the right device. *Circulation.* 2010;122(16):1629–1636.

S42. Spatz ES, Ginsburg GS, Rumsfeld JS, Turakhia MP. Wearable digital health technologies for monitoring in cardiovascular medicine. *N Engl J Med.* 2024;390(4):346–356.

S43. Avram R, Ramsis M, Cristal AD, Nathan V, Zhu L, Kim J, et al. Validation of an algorithm for continuous monitoring of atrial fibrillation using a consumer smartwatch. *Heart Rhythm.* 2021;18(9):1482–1490.

S44. Isakadze N, Martin SS. How useful is the smartwatch ECG? *Trends Cardiovasc Med.* 2020;30(7):442–448.

S45. Blandford A, Wesson J, Amalberti R, AlHazme R, Allwihan R. Opportunities and challenges for telehealth within, and beyond, a pandemic. *Lancet Glob Health.* 2020;8(11):e1364–e1365.

S46. Pfeiffer D, Jung W, Fehske W, Korte T, Manz M, Moosdorf R, et al. Complications of pacemaker-defibrillator devices: diagnosis and management. *Am Heart J.* 1994;127(4 Pt 2):1073–1080.
