# Supplementary material for: Understanding patient preferences, experiences and engagement with ambulatory heart rhythm monitoring: a scoping review
Source: BMJ Open. 2026 May 8;16(5):e110631. doi: 10.1136/bmjopen-2025-110631 (PMC13157736; doi:10.1136/bmjopen-2025-110631)
Supplement: Supplementary file 2 [file bmjopen-2025-110631.reviewer_comments.pdf]

PEER REVIEW HISTORY

BMJ Open publishes all reviews undertaken for accepted manuscripts. Reviewers are asked to complete a checklist review form and are provided with free text boxes to elaborate on their assessment. These free text comments are reproduced below.

ARTICLE DETAILS

Title (Provisional)

Understanding patient preferences, experiences and engagement with ambulatory heart rhythm monitoring: a scoping review

Authors

Bennett, Michael Robert James; Chaudhry, Bill; Norman, Gill; Robinson, Tomos; Ternent, Laura; Coats, Louise

---

VERSION 1 - REVIEW

**Reviewer**	**1**
**Name**	**Teh, Andrew**
**Affiliation**	**Austin Health**
**Date**	**05-Nov-2025**
**COI**	**None**

The current paper presents a scoping review aimed at describing the patient experience and compliance with a variety of ambulatory ECG monitoring technologies. Patient clinical characteristics, demographics, education and expectations influenced their engagement with devices. Conversely, skin irritation and method of activation were device factors.

The clinical question initially seemed to relate to the use of ambulatory monitoring for detection of AF to enable accurate diagnosis and institution of treatments in the context of patient-driven preferences. The review appears comprehensive, however, the way in which it has been presented makes for difficult reading. The scope has included studies using these devices for a variety of reasons, also making the data presented difficult to follow. Later, the authors pose the questions as being “What are patient preferences for ambulatory heart rhythm monitoring and what factors affect engagement with their use?”. In another paragraph, the authors report the aim being “to report patient preferences for AHRM and factors affecting engagement with these to better inform future care models and to identify current gaps in the literature”. The questions appear relevant as patients are more commonly being involved directly in obtaining ambulatory data. However, each of these questions appear slightly different, and perhaps this is why the authors results, discussion and conclusion could not clearly answer these.

43 studies including 5,800 patients average age 65 years between 2008 and 2024 were included. Technologies analysed included Holter monitors, patch devices, single-lead ECG devices, external and internal loop recorders, smart watches, other wearable devices and implantable pacemakers or defibrillators. The most common reason for monitoring was for investigation of symptoms or AF surveillance.

Because of the breadth of studies included, the authors have not been able to distil which devices might be appropriately recommended/preferred by patients and clinicians for specific conditions. Although patient preference should be a key consideration in the choice of ambulatory monitoring, the clinician needs to be able to determine which options are best based on the clinical question being posed

Many of the technologies included are available direct to the consumer, often without prior instruction on the technology, as much of it is available direct to the consumer. This point has not been discussed at all in the manuscript.

Clinical and demographic factors:

The authors report patients with comorbidities such as renal disease had additional challenges, some of which were potentially due to poor education such as fear of implantable loop recorders interfering with dialysis. Surprisingly, with education, older patients were more consistent users of technology.

It is unclear what the authors are inferring when reporting a lower likelihood of ICD activation when required (these devices are automated, and do not require manual patient input)

There were socioeconomic contributions with rural patients having up to 18% transmissions lost. The authors could discuss ways to overcome this.

Under experience and preferences, 60-70% of patients with ILR reported feeling safter within 6 mths of implant, however, 20% felt worse or more anxious. Health anxiety over readings is becoming a huge burden on patients and clinicians, with automated alerts from smart watches being generated without clinician oversight. This requires discussion.

Overall, patient factors included the presence of comorbidities, prior knowledge, expectations and level of support. In particular, older adults displayed good adherence after education. Patients reported favouring face to face over video/phone review and reported lack of communication/information/education as an issue.

Devices factors included invasiveness (eg ILR), comfort (skin irritation) and ability to integrate the choice of device into daily life (negative for Holter monitoring vs skin patch or smart watch).

In the discussion section, the authors discuss the preference of physicians for ECG over PPG alerts. The authors do not appear to have a poor understanding of the available literature

validating such technologies for AF diagnosis, with limited validation outside of the manufacturer sponsored studies. Based on the current lack of PPG validation, there would be few clinicians willing to start AF management based solely on PPG data, which has been consistently demonstrated to be of lower accuracy than ECG data for AF management. In addition, in order to provide accurate information on the data being reviewed by the patient and clinician, validation studies are key to allow clinicians to implement actions.

Reviewer **2**

Name **Stock, Stephanie**

Affiliation **Universitat zu Koln Institut fur Gesundheitsokonomie und Klinische Epidemiologie**

Date **09-Dec-2025**

COI **None**

Overall, this scoping review targeting patient preferences for ambulatory heart rhythm monitoring by Bennett et al. provides valuable insights for clinicians and readers alike. The manuscript is easy to follow and the conclusions seem justified. However, some issues should be addressed.

Major concerns:

1. Reading the title of the article which refers to “patient preferences” the reader expects a more specific review. The review apparently targets not only preferences but also other, different concepts which should be addressed by a broader framing of the title (patient preference is based on another concept than patient experiences or patient behavior).

2. In addition to a broader title the concepts the review targets need to be outlined and to some extent categorized. This could be done either in the introduction (e.g. when describing the inclusion criteria) or as a result of the review.

3. The authors describe they have omitted an assessment of study quality and instead have provided levels of evidence (LoE) which is correct for a scoping review. However, the review lacks the interpretation of the study results with regard to the LoE. For example, there are only two primary studies classified as LoE 1b (56 + 75) but these were not highlighted in the review. Why not? In addition, studies with LoE 2 have more significance than those with level 3 and should be considered as such. Summaries and interpretations would benefit from the consideration of LoE of the included studies.

4. Subchapter “Methodology of Studies included”: should be much more specific, e.g. were there any real preference studies (e.g., discrete choice experiments, studies using CV) or which concepts (e.g. experience, acceptance, preference) were addressed how often?

5. Section ‘Results’: the sub-sections are somewhat superficial and could include more details (e.g., how many of the studies have analyzed the outcomes presented in the respective section).

Minor concerns

Page 6, second sentence: put percentage of emergency admissions due to arrythmia into perspective (e.g. making it the nth most admission reason in England).

Page 8, first paragraph, second word: typo “the”

Page 9, line 52/53: it should be “were screened”

Page 9: Pre-registration dated after date of search conducted

Page 9: why 25 articles were screened from two reviewers but the remaining ones from one?

Page 9: the search is a little bit outdated (December 2024). Update recommended.

Page 13, line 12-14: numeration includes only 42 studies (page 12 and figure 1: 43)

Page 16: The subchapter “Impact of monitoring on ADL…” reads as a new research question that has not been stated before. I assume you want to show that the influence of monitoring devices on ADL can in turn affect the engagement and satisfaction with the devices but this could be outlined clearer.

Section ‘Results’: for all citations and results presented, present the reference of the study the statement was taken from (e.g. “clinical and demographic factors”, first paragraph).

General: distinction between methods of patient involvement (i.e. automatic data collection vs. patient initiation) is merely mentioned but not further investigated.

Discussion: statements/conclusions could benefit from referring more to the included studies.

Appendix: for supplementary table 3 a column could be added showing what paper has analyzed what outcome/what result you have used for the factors you identified (e.g. clinical patient factor; general device factor; etc.).

What is the second reference list for?

VERSION 1 - AUTHOR RESPONSE

Reviewer: 1
Dr. Andrew Teh, Austin Health

Comment 1

The current paper presents a scoping review aimed at describing the patient experience and compliance with a variety of ambulatory ECG monitoring technologies. Patient clinical characteristics, demographics, education and expectations influenced their engagement with devices. Conversely, skin irritation and method of activation were device factors.

Response

Thank you for this summary. We are pleased that the scope and key findings of the review have been accurately reflected.

Comment 2

The clinical question initially seemed to relate to the use of ambulatory monitoring for detection of AF to enable accurate diagnosis and institution of treatments in the context of patient-driven preferences. The review appears comprehensive, however, the way in which it has been presented makes for difficult reading. The scope has included studies using these devices for a variety of reasons, also making the data presented difficult to follow.

Response

We recognise that the Introduction primarily uses examples from atrial fibrillation (AF) to describe challenges in arrhythmia care, as it is the most extensively studied arrhythmia, which could give the impression that the review focuses on AF-related care. However, the review addresses ambulatory heart rhythm monitoring across all adult populations and indications, reflecting the diversity of its use.

To improve clarity, we have revised the wording in the Introduction to reflect this broader scope (page 6, lines 15 and 26). The scope is explicitly stated at the end of the Introduction (page 7, lines 7–9) and reiterated in the Methods, where we specify inclusion of monitoring for cardiac arrhythmia of any cause (page 8, lines 5–8).

To further improve clarity of presentation, study characteristics are presented cumulatively in the Results (page 12, lines 2–21), alongside visual summaries (Figures 2 and 3) and detailed study-level data in the supplementary material (Supplementary Data Tables 3 and 5). This provides information on the populations and contexts of individual studies, supporting understanding of how each contributes to the overall synthesis.

Comment 3

Later, the authors pose the questions as being “What are patient preferences for ambulatory heart rhythm monitoring and what factors affect engagement with their use?”. In another paragraph, the authors report the aim being “to report patient preferences for AHRM and factors affecting engagement with these to better inform future care models and to identify current gaps in the literature”. The questions appear relevant as patients are more commonly being involved directly in obtaining ambulatory data. However, each of these questions appear slightly different, and perhaps this is why the authors results, discussion and conclusion could not clearly answer these.

Response

Thank you for this important observation. We have revised the manuscript to ensure a single, consistent research question and aligned aims throughout (Introduction, page 7, lines 6–10; Methods, page 8, lines 10–12 and 14–18; page 10, lines 4–5). We have also clarified

the wording and scope of the research question and objectives in the Methods (page 8, lines 10–18) make it clear for the reader what the purpose of the study is and ensure that the Results, Discussion and Conclusion directly address these.

Comment 4

43 studies including 5,800 patients average age 65 years between 2008 and 2024 were included. Technologies analysed included Holter monitors, patch devices, single-lead ECG devices, external and internal loop recorders, smart watches, other wearable devices and implantable pacemakers or defibrillators. The most common reason for monitoring was for investigation of symptoms or AF surveillance.

Response

Thank you for this summary. We are pleased that the characteristics of the included studies and the scope of technologies and populations have been clearly reflected.

Comment 5

Because of the breadth of studies included, the authors have not been able to distil which devices might be appropriately recommended/preferred by patients and clinicians for specific conditions. Although patient preference should be a key consideration in the choice of ambulatory monitoring, the clinician needs to be able to determine which options are best based on the clinical question being posed

Response

We agree that device selection should be guided by the clinical question, with patient preference also playing an important role. As this is a scoping review, our aim was to map existing evidence across devices and contexts rather than provide device-specific recommendations. We have clarified this in the Introduction (Page 7, lines 6-7) and Methods (page 8, lines 5–18). This review supports clinical decision-making by improving understanding of patient preferences and factors influencing engagement with monitoring, which can be considered alongside clinical indication and guideline-based care.

Comment 6

Many of the technologies included are available direct to the consumer, often without prior instruction on the technology, as much of it is available direct to the consumer. This point has not been discussed at all in the manuscript.

Response

We have now expanded the Discussion to explicitly address direct-to-consumer monitoring technologies and the implications of their use without prior instruction, including impacts on patient understanding, engagement and healthcare utilisation (page 19, lines 22–32). We also highlight the limited evidence in this area and the need for further research to better understand these impacts (Discussion, page 19, lines 28–32).

Comment 7

Clinical and demographic factors:
The authors report patients with comorbidities such as renal disease had additional challenges, some of which were potentially due to poor education such as fear of implantable loop recorders interfering with dialysis. Surprisingly, with education, older patients were more consistent users of technology.

Response

We are encouraged that the findings on clinical and demographic factors, including the influence of education and engagement across patient groups, are clearly recognised.

Comment 8

It is unclear what the authors are inferring when reporting a lower likelihood of ICD activation when required (these devices are automated, and do not require manual patient input)

Response

We clarify that “ICD activation” refers to patient engagement with remote monitoring processes (e.g. initiating transmissions or responding to alerts), rather than automated device-delivered therapy. This has now been rephrased in the manuscript (Table 1, page 24, under ICD/PPM, Age, Clinical and Demographic factors).

Comment 9
There were socioeconomic contributions with rural patients having up to 18% transmissions lost. The authors could discuss ways to overcome this.

Response

We have expanded the Discussion to acknowledge socioeconomic and geographic barriers, including connectivity limitations in rural settings. We now outline potential mitigation strategies, such as improving digital infrastructure, using alternative data transmission methods where connectivity is limited (e.g. offline or delayed upload), and adapting follow-up models to local contexts (page 19, lines 16–20).

Comment 10

Under experience and preferences, 60-70% of patients with ILR reported feeling safter within 6 mths of implant, however, 20% felt worse or more anxious. Health anxiety over readings is becoming a huge burden on patients and clinicians, with automated alerts from smart watches being generated without clinician oversight. This requires discussion.

Response

We have expanded the Discussion to further address health anxiety associated with monitoring, including the impact of automated alerts from wearable devices without clinician oversight and the potential burden on patients and healthcare services (page 19, lines 24-28. We also highlight the need for appropriate education, feedback and system design to mitigate these effects (page 20, lines 1-3).

Comment 11

Overall, patient factors included the presence of comorbidities, prior knowledge, expectations and level of support. In particular, older adults displayed good adherence after education. Patients reported favouring face to face over video/phone review and reported lack of communication/information/education as an issue.

Devices factors included invasiveness (eg ILR), comfort (skin irritation) and ability to integrate the choice of device into daily life (negative for Holter monitoring vs skin patch or smart watch).

Response

This summary accurately reflects our findings. The importance of education, support and communication in shaping patient engagement is clearly highlighted, alongside key device-related factors such as invasiveness, comfort and the ability to integrate monitoring into daily life.

Comment 12

In the discussion section, the authors discuss the preference of physicians for ECG over PPG alerts. The authors do not appear to have a poor understanding of the available literature validating such technologies for AF diagnosis, with limited validation outside of the manufacturer sponsored studies. Based on the current lack of PPG validation, there would be few clinicians willing to start AF management based solely on PPG data, which has been consistently demonstrated to be of lower accuracy than ECG data for AF management. In addition, in order to provide accurate information on the data being reviewed by the patient and clinician, validation studies are key to allow clinicians to implement actions.

Response

We agree that ECG confirmation following PPG alerts is necessary and that robust validation is essential to support accurate clinical decision-making and patient understanding. We do not present PPG as a standalone diagnostic modality or make device-specific recommendations beyond guideline-based care; rather, clinician perspectives are considered as one of several factors influencing device selection. The relevant section of the manuscript has been revised to clarify this (page 21, line 27 to page 22, line 2).

Reviewer: 2
Prof. Stephanie Stock, Universitat zu Koln Institut fur Gesundheitsokonomie und Klinische Epidemiologie

Comment 1
Overall, this scoping review targeting patient preferences for ambulatory heart rhythm monitoring by Bennett et al. provides valuable insights for clinicians and readers alike. The manuscript is easy to follow and the conclusions seem justified. However, some issues should be addressed.

Response

Thank you for your positive and constructive feedback. We are pleased that the review was found to be clear and the conclusions appropriate, and we have addressed the points raised below.

Comment 2

1. Reading the title of the article which refers to "patient preferences" the reader expects a more specific review. The review apparently targets not only preferences but also other, different concepts which should be addressed by a broader framing of the title (patient preference is based on another concept than patient experiences or patient behavior).

Response

We agree that the review encompasses patient preferences alongside the related concepts of experience and engagement. We have therefore revised the title to better reflect the breadth of the review and ensure alignment with the scope of the study – "Understanding patient preferences, experiences and engagement with ambulatory heart rhythm monitoring: a scoping review".

Comment 3

2. In addition to a broader title the concepts the review targets need to be outlined and to some extent categorized. This could be done either in the introduction (e.g. when describing the inclusion criteria) or as a result of the review.

Response

We agree that outlining and more explicitly categorising the concepts within the paper would be helpful for the reader. We have therefore added a section to the Methods, "Conceptual focus and synthesis", which provides greater and clearer information on concept and organisation of outcomes (page 10, lines 3-14). In addition, the concepts of the scoping review are also defined through a Population–Concept–Context framework (Methods, page 8, lines 3-8) and outlining of the research question and specific objectives (Methods, page 8, lines 10-18).

Comment 4

3. The authors describe they have omitted an assessment of study quality and instead have provided levels of evidence (LoE) which is correct for a scoping review. However, the review lacks the interpretation of the study results with regard to the LoE. For example, there are only two primary studies classified as LoE 1b (56 + 75) but these were not highlighted in the review. Why not? In addition, studies with LoE 2 have more significance than those with level 3 and should be considered as such. Summaries and interpretations would benefit from the consideration of LoE of the included studies.

Response

We acknowledge that studies with higher levels of evidence were not preferentially weighted or highlighted in the synthesis. This approach is appropriate for a scoping review and is consistent with PRISMA-ScR and JBI guidance, which prioritise mapping the extent, range, and nature of evidence rather than formally weighting findings by study quality or level of evidence. In this review, higher-level studies (e.g. LoE 1–2) often assessed patient preference or experience as secondary outcomes, whereas observational and qualitative studies more directly addressed these concepts. In particular, qualitative studies provide important contextual and experiential insights that are not captured through quantitative

designs. As such, levels of evidence were used descriptively to characterise the evidence base rather than to determine the prominence of findings. We have clarified this rationale in the Methods (page 10, lines 15–22).

Comment 5

4. Subchapter "Methodology of Studies included": should be much more specific, e.g. were there any real preference studies (e.g., discrete choice experiments, studies using CV) or which concepts (e.g. experience, acceptance, preference) were addressed how often?

Response

We have clarified the concepts addressed and study methodologies in the "Methodology of included studies" section, in the Results, including that no formal stated-preference methods were identified (Results, page 13, lines 7–9). The subsequent "Themes and interpretation" section outlines how experience, acceptance and preference are captured within the key themes identified within the study, including the number of studies contributing to each theme and subtheme (page 13, lines 10–21).

Comment 6

5. Section 'Results': the sub-sections are somewhat superficial and could include more details (e.g., how many of the studies have analyzed the outcomes presented in the respective section).

Response

We have added a section within the "Themes and Interpretation" of the results which quantifies the relative contribution of studies to each sub-section (page 13, lines 10–21). We have also added a supplementary table (Supplementary Table 5) indicating exactly which subsection each study contributed to, enhancing transparency regarding the evidence underpinning each theme.

Comment 7

Page 6, second sentence: put percentage of emergency admissions due to arrythmia into perspective (e.g. making it the nth most admission reason in England).

Response

Thank you for this suggestion. While we agree that additional context would be helpful, detailed categorisation of emergency admissions by arrhythmia relative to other causes is not readily available in published datasets. The reported figure was derived from aggregated national data, and further stratification was not feasible. We have therefore retained the original wording.

Comment 8

Page 8, first paragraph, second word: typo "the"

Response

We have corrected this typo.

Comment 9

Page 9, line 52/53: it should be "were screened"

Response

We have corrected this typo.

Comment 10

Page 9: Pre-registration dated after date of search conducted

Response

We have corrected this typo.

Comment 11

Page 9: why 25 articles were screened from two reviewers but the remaining ones from one?

Response

An initial subset of 25 articles was double-screened to ensure consistency in the application of inclusion criteria between reviewers. As agreement was high, the remaining records were screened by a single reviewer, with any uncertainties reviewed and resolved through discussion with a second reviewer. This approach was chosen to balance the practicalities of screening studies and funding limitations for the scoping review.

Comment 12

Page 9: the search is a little bit outdated (December 2024). Update recommended.

Response

We have updated the literature search across all platforms used in the original strategy and the dates of when searches were performed are included in the Methods (page 8, line 27-28). This identified eight additional original research articles that were subsequently included in the scoping review. Four of these studies evaluated smartwatch technologies and further highlighted both the potential benefits and challenges associated with their use, including increased healthcare utilisation and heightened anxiety. Overall, these studies were consistent with the existing evidence base, reinforcing that patients tend to prefer devices that minimise disruption to daily life and provide meaningful feedback.

Comment 13

Page 13, line 12-14: numeration includes only 42 studies (page 12 and figure 1: 43)

Response

We have corrected this and ensured it is accurate across the manuscript.

Comment 14

Page 16: The subchapter "Impact of monitoring on ADL…" reads as a new research question that has not been stated before. I assume you want to show that the influence of monitoring devices on ADL can in turn affect the engagement and satisfaction with the devices but this could be outlined clearer.

Response

Thank you for this helpful observation. We have revised this section to clarify that the impact of monitoring on activities of daily living is a factor influencing patient experience, engagement and satisfaction, rather than a separate research question (page 15, lines 21-22).

Comment 15

Section 'Results': for all citations and results presented, present the reference of the study the statement was taken from (e.g. "clinical and demographic factors", first paragraph).

Response

We have reviewed the results section ad ensured where a statement appears (between parentheses) that the corresponding study is referenced in isolation. Sub-sections in the Results where this has been amended include Clinical and demographic factors (page 13, line 28) , Education and expectations (page 14, line 23 and line 24), Impact on daily living and healthcare interaction (page 16, line 9) and Specific factors (page 16, line 27 and line 30).

Comment 16

General: distinction between methods of patient involvement (i.e. automatic data collection vs. patient initiation) is merely mentioned but not further investigated.

Response

We agree that the distinction between passive (automatic) and active (patient-initiated) monitoring is important. However, the extent to which the patient was involved was not consistently reported across included studies. We have highlighted this in the Discussion (page 20, lines 24-26).

Comment 16

Discussion: statements/conclusions could benefit from referring more to the included studies.

Response

We have revised the Discussion to more consistently reference the included studies in support of key statements and conclusions, improving transparency and alignment with the Results (page 19, line 16 and line 28; page 20, line 11, line 23 and line 28).

Comment 17

Appendix: for supplementary table 3 a column could be added showing what paper has analyzed what outcome/what result you have used for the factors you identified (e.g. clinical patient factor; general device factor; etc.).

Response

We have added an a further supplementary table (Supplementary Data Table 5) which shows how each study contributed to both the themes and specific subthemes

Comment 18

What is the second reference list for?

Response

The second reference list related to sources cited within the supplementary material for Supplementary Data Table 1. We have now combined all references into a single reference list for clarity.
